# Multiple-Molecule Drug Design Based on Systems Biology Approaches and Deep Neural Network to Mitigate Human Skin Aging

**DOI:** 10.3390/molecules26113178

**Published:** 2021-05-26

**Authors:** Shan-Ju Yeh, Jin-Fu Lin, Bor-Sen Chen

**Affiliations:** Laboratory of Automatic Control, Signal Processing and Systems Biology, Department of Electrical Engineering, National Tsing Hua University, Hsinchu 30013, Taiwan; m793281@gmail.com (S.-J.Y.); sweettofu531@gmail.com (J.-F.L.)

**Keywords:** skin aging, oxidative stress, aging progression mechanism, genome-wide genetic and epigenetic network (GWGEN), systems medicine design, multiple-molecule drug

## Abstract

Human skin aging is affected by various biological signaling pathways, microenvironment factors and epigenetic regulations. With the increasing demand for cosmetics and pharmaceuticals to prevent or reverse skin aging year by year, designing multiple-molecule drugs for mitigating skin aging is indispensable. In this study, we developed strategies for systems medicine design based on systems biology methods and deep neural networks. We constructed the candidate genomewide genetic and epigenetic network (GWGEN) via big database mining. After doing systems modeling and applying system identification, system order detection and principle network projection methods with real time-profile microarray data, we could obtain core signaling pathways and identify essential biomarkers based on the skin aging molecular progression mechanisms. Afterwards, we trained a deep neural network of drug–target interaction in advance and applied it to predict the potential candidate drugs based on our identified biomarkers. To narrow down the candidate drugs, we designed two filters considering drug regulation ability and drug sensitivity. With the proposed systems medicine design procedure, we not only shed the light on the skin aging molecular progression mechanisms but also suggested two multiple-molecule drugs for mitigating human skin aging from young adulthood to middle age and middle age to old age, respectively.

## 1. Introduction

Being the largest organ of the human body, the skin shows aging with biological age. Many people, especially female, like to spend money on cosmetics and pharmaceuticals regularly for preventing or reversing skin aging. Thus, changes in human skin caused by aging are important issues for both the pharmaceutical and cosmetic sectors worldwide [1]. Additionally, increasing life expectancy in developed countries reveals advancing age as the primary risk factor for numerous diseases [2]. Elder people tend to have dryness, itch, dyspigmentation, wrinkles, as well as benign and malignant tumors on skin. Under these worse conditions, they would feel sleep deprivation leading to having weakened immunity and getting infection. Hence, keeping our skin health promotes healthy aging [3]. Furthermore, identifying interventions, which are able to ameliorate skin aging progression, to delay, prevent or lessen age-related diseases is worth studying.

Human skin provides a primary protective barrier, routinely shielding us from allergens, microbes, and other environmental assaults, including solar ultraviolet (UV) irradiation, heat, infection, water loss, and injury. Skin aging is a complex process leading to the decrement of cutaneous functions and structures with time. Impaired epidermal barrier function, decline in resistance to infections and regenerative potential, and impairment of mechanical properties like loss of extensibility and elasticity are the essential biomarkers of human skin ageing [4]. In general, skin aging can be regarded as two different processes. The first one is intrinsic aging, which is caused by biological age. The second one is extrinsic aging, which arises from solar UV exposure. The extrinsic factors contain the exposure under UV radiation and pollution, and poor nutrition resulting in alterations of DNA, RNA and protein in skin cells. The clinical manifestation of intrinsic aging is characterized by age spots, laxity, wrinkles, sagging, dryness, itchy, and the lower type I and III fibrillar collagens leading to dermal atrophy [5].

MicroRNAs (miRNAs) are a group of small noncoding RNAs, owning the post-transcriptional regulation ability to control gene expression negatively. Meanwhile, they are found to involve in many biological processes, such as epidermal development, proliferation, differentiation [6,7,8], inflammatory responses, immune regulation and wound healing in human skin [9,10]. Although we know that miRNAs might be a key player in the age-associated change, studies about age-related miRNAs in human skin remain limited [11]. As for long noncoding RNAs (lncRNAs), they are another type of noncoding RNA with >200 nucleotides. One review paper has summarized age-related lncRNAs and elucidated their roles in different aging process [12]. Since lncRNAs have versatile functions including gene regulation, chromatin structure modulation, genomic imprinting, cell growth and differentiation, and embryonic development, the dysregulated expression of lncRNAs may cause age-related diseases and disorders [13]. Recently, lncRNAs are regarded as potential targets for antiaging therapies [14]. Moreover, the well-known epigenetic modifications are DNA methylation and histone post-transcriptional modifications, including methylation, acetylation, ubiquitination, and phosphorylation. The accumulation of epigenetic alternations may not only contribute to skin aging but also promote malignant transformation [15,16]. H19, an epigenetic regulatory RNA, has been demonstrated to positively affect cell growth and proliferation and delay senescence [17]. With epigenetic silencing on LMNA, which is one of progeroid genes, we could observe a corresponding malignant transformation [18].

For the purpose of investigating skin aging process, researchers tried to identify the influence of microenvironment and epigenetic change on skin aging and put focus on some specific proteins, such as members of the collagen family, or cellular functions. However, the definitions of young skin and older skin are not fixed, that is, there is no definition of age range for young people and old people, respectively. Therefore, most studies compared young and old people with great differences in the research of skin aging progression. Although these studies proposed plenty of credible skin aging-associated theories and experimental results, the genomewide molecular progression mechanism of skin aging was unknown since the restriction of experimental methods and the attention to specific proteins and cellular functions. Moreover, although pharmacological interventions may prove to ameliorate the effect of aging on humans, the prohibitive expansion of treating healthy individuals in clinical trials over a long duration becomes a crucial difficulty in developing new drugs. On the contrary, repurposing drugs, which have been already approved for specific diseases, or those have been passed their safety tests but failed against their original indication, is more feasible than targeting aging itself with new drugs [19,20,21].

In recent years, pharmaceutical scientists put a lot of efforts into novel drug development based on the knowledge of existing drugs [22,23]. By performing in vitro search for drug discovery, researchers could identify interaction between drugs and targets (e.g., genes). However, due to the high cost and time consuming work, we could not conduct in vitro research most of the time. Instead, virtual screening in silico, selecting possible candidates first and verifying them in wet laboratory offer alternatives to us [24]. In general, docking simulation and machine learning method are considered to be two main approaches for in silico prediction of drug–target interaction [25]. For docking simulation, the process would be limited if the 3D structure of the protein is unknown [26]. To deal with this issue, chemogenomic methods, namely feature-based methods, transform drugs and targets into sets of descriptors (e.g., feature vector) allowing machine learning models to make prediction of drug–target interactions [27]. Chen at al. reviewed machine learning methods and databases that used chemogenomic approaches for drug–target interaction prediction [28]. Except for traditional machine learning methods, the deep neural network has been employed in drug–target interaction prediction as well, such as deep belief neural networks [29], convolutional neural network [30], and multilayer perceptron [31,32].

In this study, we define different age intervals for each stage of skin aging. We build the candidate genome-wide genetic and epigenetic network (GWGEN) containing a candidate protein–protein interaction network (PPIN) and a candidate gene regulatory network (GRN) by big database mining. Moreover, it can be represented by a binary matrix. Assisted with microarray data of human skin, the false positives from the candidate GWGEN are pruned away by the system identification method and system order detection scheme. By doing so, we obtain real GWGENs of young-adult, middle-aged, and elderly skin aging as shown in Figures S1–S3, respectively. However, real GWGENs are still complex. Therefore, we further extract core GWGENs from real GWGWNs by the principal network projection (PNP) method. Based on the rank of projection values, we could obtain the core signaling pathways in respect of KEGG pathways to investigate skin aging molecular progression mechanisms for each stage of skin aging. To identify essential biomarkers in core signaling pathways, we refer to GenAge [33], which contains genes involved in human aging progression, and the Connectivity Map (CMap) [34] dataset to find overlap nodes being drug targets. To explore the potential candidate drugs toward our identified biomarkers, we trained a deep neural network of drug–target interactions in advance. By applying it, we could predict potential candidate drugs, which holds higher interaction probability to the identified biomarker. Afterwards, we designed two filters considering drug regulation ability and drug sensitivity to narrow down the candidate drugs. Consequently, we propose two potential multiple-molecule drugs i.e., niridazole, liothyronine, decitabine, pinacidil, and allantoin for mitigating skin aging from young adulthood to middle age; allantoin, diclofenac, mepyramine, resveratrol, and azathioprine for mitigating skin aging from middle age to old age.

## 2. Results

**Figure 1:**
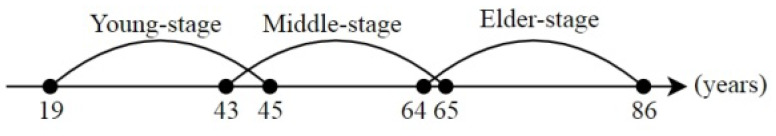
Skin aging stages. The figure denotes age intervals for each stage of skin. The young-adult, middle-aged, and elderly stage of skin are defined as 19 to 45 years old, 43 to 65 years old, and 64 to 86 years old, respectively.

**Figure 2:**
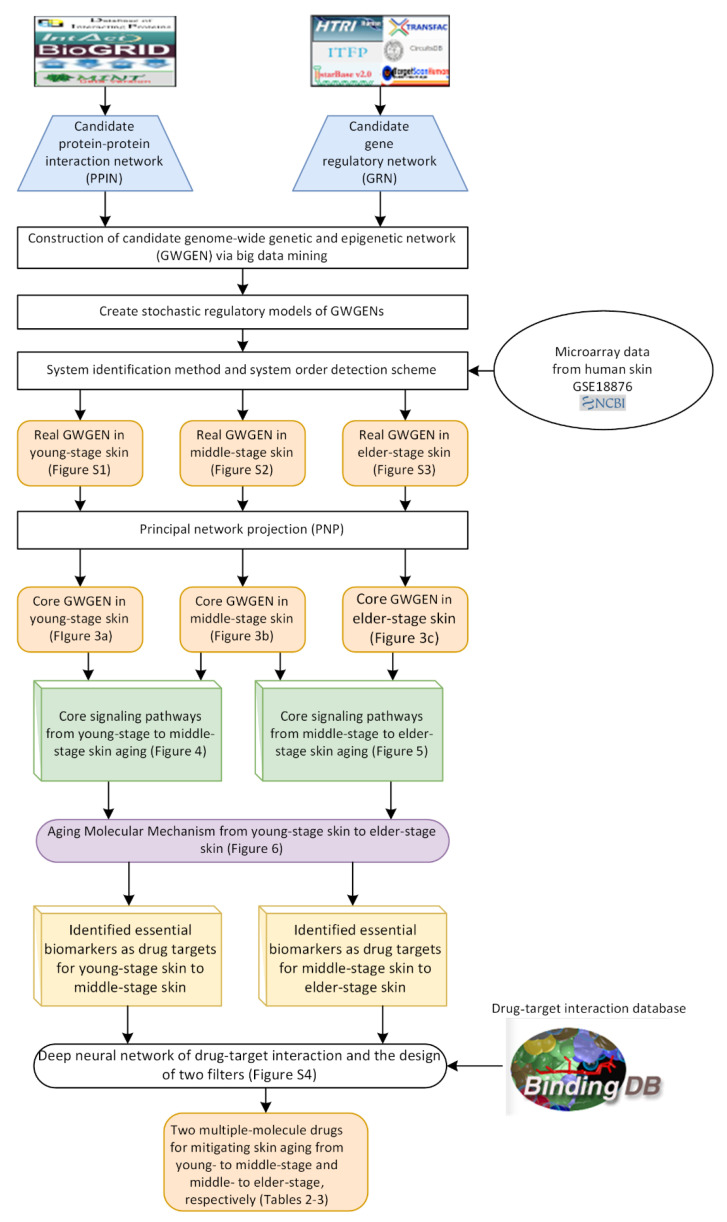
Reseasrch flowchart of systems medicine design for human skin aging. Flowchart of using systems biology methods to construct the candidate GWGEN, real GWGENs, core GWGENs, and core signaling pathways to find skin aging progression mechanisms for identifying essential biomarkers. After obtaining the essential biomarkers, we applied trained a deep neural network of drug–target interactions to predict the potential candidate drugs holding higher probability. To narrow down the candidate drugs, we considered drug regulation ability by querying the CMap dataset and drug sensitivity by referring to the sensitivity dataset from DepMap portal. Consequently, we proposed two multiple-molecule drugs to mitigate the skin aging from young-adult to middle-aged and middle-aged to elderly-stage.

**Figure 3:**
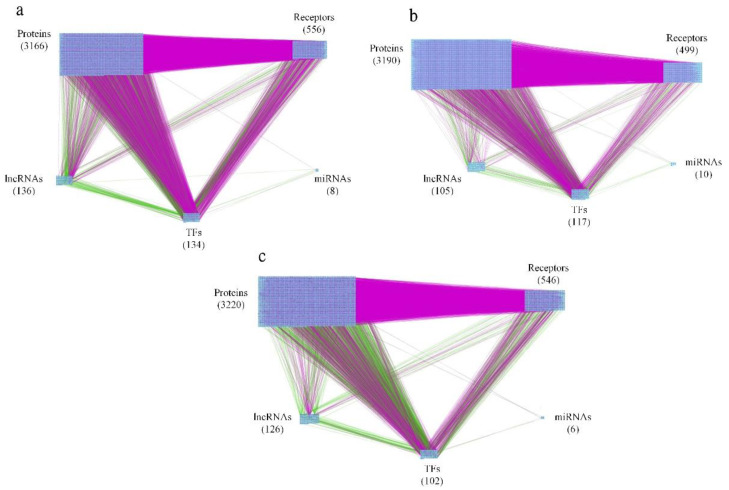
The core genomewide genetic and epigenetic networks (GWGEN). (**a**–**c**). (**a**) The core GWGEN of young-adult skin. The purple lines denote protein–protein interactions (PPIs). The green lines indicate transcriptional regulations by TFs and lncRNAs. The black lines represent post-transcriptional regulations by miNRAs. The total number of receptors, proteins, lncRNAs, TFs and miRNAs are 556, 3166, 136, 134 and 8, respectively. (**b**) The core GWGEN of middle-aged skin. The PPIs are in purple. The regulations from TFs and lncRNAs are in green. The black lines stand for the post-transcriptional regulations by miRNAs. The total number of receptors, proteins, lncRNAs, TFs and miRNAs are 499, 3190, 105, 117 and 10, respectively. (**c**) The core GWGEN of elderly-stage skin. The PPIs are shown in purple lines; regulations by TFs and lncRNAs are denoted in green; regulations from miRNAs are in black. The total number of receptors, proteins, lncRNAs, TFs and miRNAs are 546, 3220, 126, 102 and 6, respectively.

For the purpose of analyzing molecular progression mechanisms in human skin aging, extracting core signaling pathways from each core GWGEN becomes an essential issue. We defined three skin aging stages, including young-adult, middle-aged, and elderly human skin as shown in Figure 1. The research flowchart in Figure 2 shows how to construct the candidate GWGEN, real GWGENs, and core GWGENs so as to extract core singling pathways and investigate molecular progression mechanisms of human skin aging. By big database mining, the candidate GWGEN containing candidate PPIN and candidate GRN was constructed. With the help of the corresponding young-adult, middle-aged, and elderly skin microarray data, we applied system identification and system order detection methods to the candidate GWGEN for obtaining real GWGENs shown in Figures S1–S3 (Supplementary Materials), respectively. The Table 1 shows the number of nodes (e.g., proteins, TFs, miRNAs, and lncRNAs) as well as the edges standing for the interaction or regulation between two nodes for the candidate GWGEN and real GWGENs. According to Table 1, compared the nodes in candidate GWGEN to the nodes in real GWGENs, one could realize that the number of nodes diminish a lot in real GWGENs, reflecting that the false positives were removed successfully by the system order detection scheme. Since the real GWGENs were still too complicated to investigate molecular progression mechanisms of human skin aging, we applied principal network projection (PNP) method and selected the top-ranked 4000 nodes with significant projection values that could reflect 85% of the real GWGENs in three stages of skin aging to obtain core GWGENs (Figure 3a–c), respectively. In addition, for the genes in core GWGENs, we used the Database for Annotation, Visualization and Integrated Discovery (DAVID) Bioinformatics Resources version 6.8 to perform enrichment analyses for each stage of skin aging as shown in Tables S1–S3, respectively. Moreover, for investigating molecular progression mechanisms of skin aging conveniently, we denoted differential core signaling pathways for young adult to middle-aged and middle-aged to elderly skin aging in respect of KEGG pathways, respectively. Based on skin aging molecular progression mechanisms, we identified essential biomarkers as drug targets for young-adult to middle-aged and middle-aged to elderly skin aging, respectively. Exploring candidate drugs toward our identified biomarkers, we trained a deep neural network of drug–target interaction in advance. We applied the trained model to predict the candidate drugs holding higher interaction probability with identified biomarkers. In order to narrow down the candidate drugs, we design two filters considering drug regulation ability and drug sensitivity. The more details will be discussed in the following sections.

### 2.1. Differential Core Signaling Pathways from Young-Adult to Middle-Aged Skin Aging

**Figure 4:**
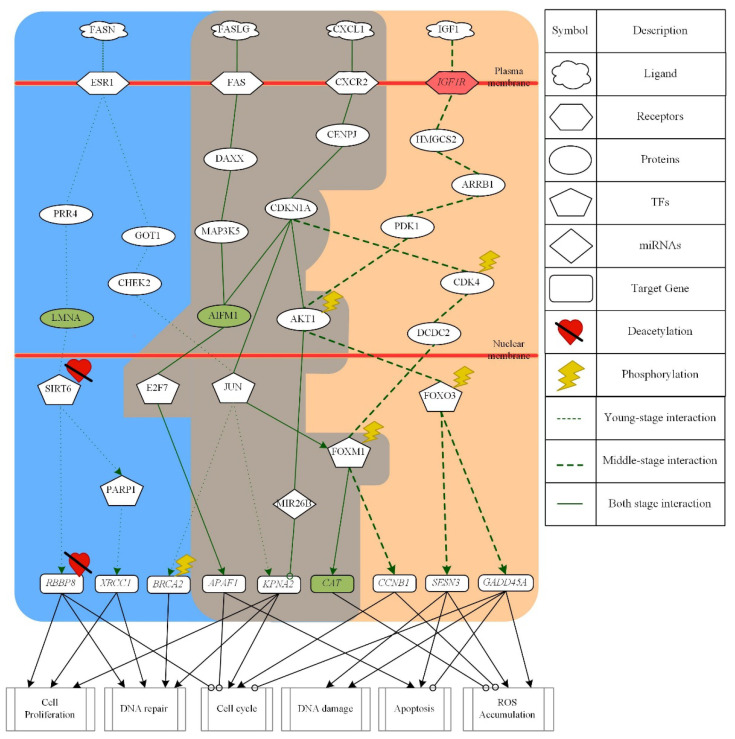
The core signaling pathways from young-adult to middle-aged skin aging. The green dot lines denote the signaling pathways in young-adult skin; the green dash lines represent the signaling pathways in middle-aged skin; the green solid lines indicate the signaling pathways in both stage; the green lines with arrow heads are upregulation (positive regulation); the green lines with circular heads are downregulation (negative regulation); the black solid lines with arrow heads mean activating cellular function; the black solid lines with circular heads mean inhibiting cellular function; the selected red target gene nodes indicate a higher gene expression in middle-aged skin compared with young-adult skin; the selected green target gene nodes indicate a lower gene expression in middle-aged skin compared with young-adult skin; the blue background shows young-adult skin; the brown background shows the overlap between young-adult and middle-aged skin; the skin color background shows middle-aged skin.

The differential core signaling pathways from young-adult to middle-aged human skin were selected and analyzed as shown in Figure 4. According to our results, in core signaling pathways of young-adult skin aging only, receptor ESR1 receives microenvironment factor FASN to activate the TF SIRT6 through signaling transduction proteins PRR4 and LMNA. The TF SIRT6 could not only downregulate target gene *RBBP8*, which was modified by deacetylation, but also activate TF PARP1 to upregulate target gene *XRCC1* to promote cell proliferation and DNA repair in young-adult skin aging only. The receptor ESR1 also regulates TF JUN through signaling transduction proteins GOT1 and CHEK2 to regulate target gene *BRCA2* and *KPNA2*. TF JUN not only activates the target gene *BRCA2*, which was modified by phosphorylation, to promote DNA repair, but also downregulates the target gene *KPNA2* to promote cell proliferation, DNA repair and cell cycle in young-adult skin aging only.

Next, in the core signaling pathways of both young-adult and middle-aged skin aging in Figure 4, the microenvironment factor FASLG was received by receptor FAS to activate TF E2F7 via signaling transduction proteins DAXX, MAP3K5 and AIFM1 to downregulate target gene *APAF1* to promote apoptosis and cell-cycle arrest. In the next pathway, the receptor CXCR2 receive microenvironment factor CXCL1 to regulate TF E2F7, TF JUN, TF FOXM1 and miRNA MIR26B. First, the TF JUN was activated through signaling transduction proteins CENPJ and CDKN1A to activate TF FOXM1. As TF FOXM1, which was modified by phosphorylation, was activated, the target gene *CAT* was upregulated to inhibit ROS accumulation in both young-adult and middle-aged skin aging. The miRNA MIR26B was activated via signaling transduction proteins CENPJ, CDKN1A and AKT1 to inhibit target gene *KPNA2* so as to promote cell proliferation. It is noted that, protein AKT1 was modified by phosphorylation. The TF E2F7 was also regulated by protein AIFM1 to downregulate target gene *APAF1* in both young-adult and middle-aged skin aging.

In the core signaling pathways of middle-aged skin aging only, the TF FOXM1 was also activated through signaling transduction proteins CENPJ, CDKN1A, CDK4 and DCDC2 when receptor CXCR2 received microenvironment factor CXCL1 in middle-aged skin aging only as shown in Figure 4. With the activation of FOXM1, the target gene *CCNB1* was upregulated to promote cell-cycle arrest and inhibit ROS accumulation. For the next pathway in middle-aged skin aging only, the microenvironment factor IGF1 was received by receptor IGF1R to regulate TF FOXO3 via signaling transduction proteins HMGCS2, ARRB1, PDK1 and AKT1. Additionally, the protein AKT1 and TF FOXO3 were modified by phosphorylation. The TF FOXO3 downregulates not only target gene *SENS3* to promote DNA damage, apoptosis, and ROS accumulation, but also target gene *GADD45A* to promote DNA damage, apoptosis, and ROS accumulation and inhibit cell-cycle arrest in middle-aged skin aging only.

In summary, when the young-adult skin aging turned into middle-aged skin aging, DNA repair ability decreases and cell cycle starts to be arrested. Thereby, ROS accumulation increases and further promotes DNA damage and apoptosis in skin cells. Additionally, these molecular progression mechanisms from young-adult to middle-aged might potentially accelerate skin aging process in elderly skin aging. According to the core signaling analyses results and considering the overlap nodes between the GenAge and CMap datasets, we identified AIFM1, CAT, IGF1R, and LMNA as essential biomarkers for preventing skin aging from young adulthood to middle-age.

### 2.2. Differential Core Signaling Pathways from Middle-Aged to Elderly Skin Aging

**Figure 5:**
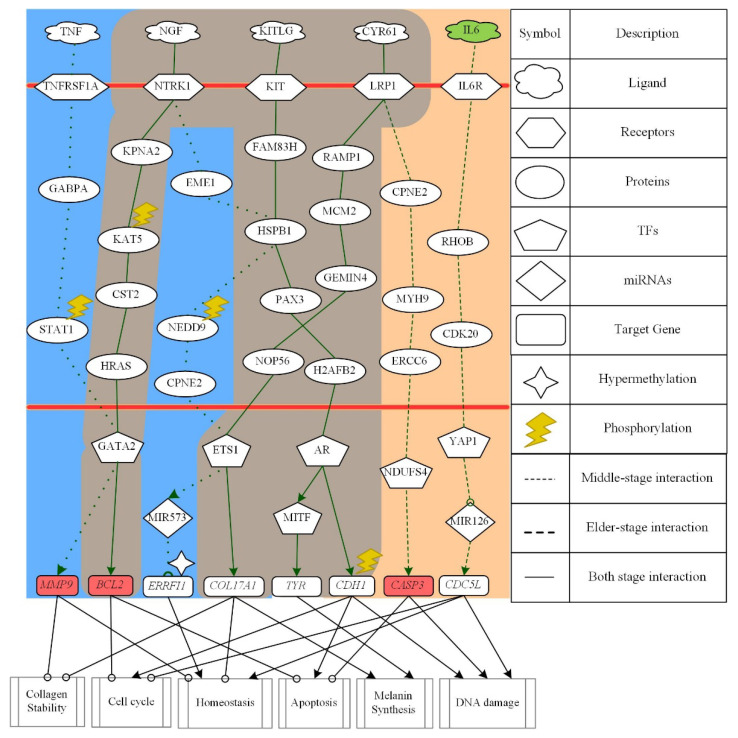
The core signaling pathways are obtained by projecting core GWGENs to KEGG pathways to investigate the aging progression mechanism from middle-aged to elderly skin aging. The green dotted lines denote the signaling pathways in middle-aged skin; the green dashed lines represent the signaling pathways in elderly skin; the green solid lines indicate the signaling pathways in both stages; the green lines with arrow heads are upregulation; the green lines with circle heads are downregulation; the black solid lines with arrow heads mean activating cellular function; the black solid lines with circle heads mean inhibiting cellular function; the selected red target gene nodes indicate a higher gene expression in elderly skin compared with middle-aged skin; the selected green target gene nodes indicate a lower gene expression in elderly skin compared with middle-aged skin; the blue background shows middle-aged skin; the brown background covers the overlap between middle-aged and elderly skin; the skin color background shows elderly skin.

The molecular progression mechanism based on differential core pathways from middle-aged skin to elderly skin aging is represented in Figure 5. In core pathways of middle-aged skin aging only, the TNF receptor superfamily member 1 alpha TNFRSF1A received microenvironment factor TNF to activate TF GATA2 through transduction proteins GABPA and STAT1 to upregulate target gene *MMP9* so as to inhibit collagen stability and skin homeostasis in middle-aged skin aging only. Note that STAT1 was modified by phosphorylation. In the next pathway, the microenvironment factor NGF was accepted by neurotrophic receptor tyrosine kinase1 NTRK1 and then transmitted the signal through transduction proteins EME1, HSPB1, NEDD9 and CPNE2 to activate TF ETS1. TF ETS1 could downregulate target gene *ERRFI1*, which was modified by hypermethylation, through activating miRNA MIR573 to promote homeostasis in middle-aged skin aging only.

Next, we focus on the core pathways of both middle-aged and elderly skin aging. In the first pathway, the receptor NTRK1 receives the microenvironment factor NGF and then transmits the signal through transduction proteins KPNA2, KAT5, CST2 and HRAS to activate TF GATA2. The protein KAT5 was modified by phosphorylation. After GATA2 was activated, target gene *BCL2* was upregulated to inhibit cell-cycle arrest and apoptosis in both middle-aged and elderly skin aging. For the second pathway, the receptor KIT could interact with the microenvironment factor KITLG to trigger TF AR through signaling transduction proteins FAM83H, HSPB1, PAX3 and H2AFB2. TF AR downregulated not only target gene *TYR* through triggering TF MITF to promote melanin synthesis, but also target gene *CDH1*, which was modified by phosphorylation, to promote cell-cycle, apoptosis and DNA damage in both middle-aged and elderly skin aging. In the third pathway, receptor LRP1 could receive the microenvironment factor CYR61 to trigger the TF ETS1 via signaling transduction proteins RAMP1, MCM2, GEMIN4 and NOP56. In both middle-aged and elderly skin aging, the TF ETS1 could negatively regulate target gene *COL17A1* to promote melanin synthesis and inhibit collagen stability and skin homeostasis.

In the core pathways of elderly skin aging only in Figure 5, CYR61/LRP1 could also trigger TF NDUFS4 through signaling transduction proteins CPNE2, MYH9 and ERCC6. The activated TF NDUFS4 might downregulate target gene *CASP3* to inhibit apoptosis and promote DNA damage. For another pathway, the microenvironment factor IL6 was accepted by receptor IL6R to trigger TF YAP1 through signaling transduction proteins RHOB and CDK20. In the elderly skin aging only, TF YAP1 activated target gene *CDC5L* through inhibiting MIR126 to inhibit cell cycle and promote skin homeostasis and DNA damage.

**Figure 6:**
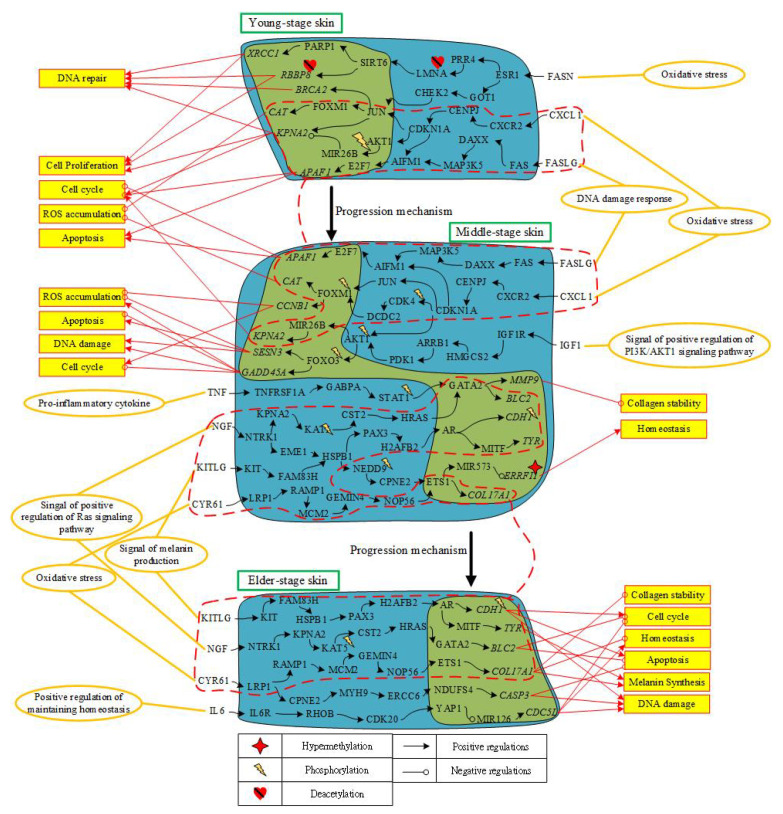
The overview of human skin aging molecular progression mechanisms from young-adult to middle-aged and then elderly skin aging. This figure summarizes the genetic and epigenetic progression mechanisms of skin aging in Figure 4 and Figure 5. The upper horizontal part is the genetic and epigenetic progression mechanism from young-adult skin to middle-aged skin; the middle part indicates the genetic and epigenetic progression mechanisms from middle-aged skin to elderly skin; the red rectangle with orange background represents cellular functions; the yellow ellipse circles are microenvironment factors; the red dash lines surround the pathways and biomarkers that appear in two consecutive stages of skin; the black arrow lines represent the protein–protein interaction or transcriptional regulation; the black lines with circle head represent inhibit or downregulation; the red arrow lines represent the genes to induce cellular function; the red lines with circle head represent the genes to repress cellular function.

In summary, for skin aging molecular progression mechanisms from middle age to old age, we found that the promotion of cell cycle process, the inhibition of apoptosis, and the damage of DNA arose in elderly skin. Furthermore, skin homeostasis and collagen stability were destroyed to cause lower immunity and epidermal thinning, that is, the increment of wrinkles. According to core signaling analyses and considering the overlap nodes between the GenAge and CMap datasets, we identified MMP9, IL6, BCL2, and CASP3 as essential biomarkers for preventing skin aging from middle age to old age. Moreover, by extracting differential core signaling pathways from young-adult to elderly skin aging, some cellular dysfunctions including proliferation, DNA repair and damage, cell-cycle arrest, apoptosis, ROS accumulation, collagen stability, skin homeostasis, and melanin synthesis are induced in the skin aging process shown in Figure 6.

### 2.3. The Application of Deep Neural Network of Drug–Target Interaction Prediction and the Design of Two Filters Considering Drug Regulation Ability and Drug Sensitivity

To explore the drug–target interaction toward our identified biomarkers, we trained a deep neural network for drug–target interaction prediction. The design framework is shown in Figure S4. The interaction dataset used for training are from BindingDB [35]. In total, there are 80,291 known drug–target interactions between 38,015 drugs and 7292 proteins. The number of unknown drug–target interactions is 19,966,109, which is greater than the known drug–target interactions. Considering the class imbalance problem, we randomly chose the number of unknown interactions and made them the same size as known interactions. We trained the model using 70% of data, including 10% of data as the validation set. The remaining 30% of data was used as the testing set. To the data preprocessing before training the model, we performed feature scaling by standardization. Assisted with principal component analyses (PCA) for dimensionality reduction, we had 1000 out of 1359 features. For the architecture of deep neural network of drug–target interaction, we used Adam as an optimizer (learning rate = 0.003) with binary cross-entropy loss. The input layer had 1000 neurons followed by 512, 256, 128, and 64 neurons of hidden layers, respectively. The output layer has one neuron. Except for using sigmoid function to the output layer, we set a nonlinear activation function ReLU for each hidden layer. Moreover, the dropout 0.5, 0.4, 0.3, and 0.1 was applied to each hidden layer, respectively. For the trained deep neural network of drug–target interaction prediction, the training accuracy, validation accuracy, and testing accuracy were 95.469%, 93.230%, and 93.077%, respectively. From the perspective of the deep neural network framework application, we used it to predict the potential candidate drugs for our identified biomarkers. When the score of candidate drug approaches one, it would be selected. In other words, the higher the score, the higher probability of interacting between the candidate drug and the identified biomarker.

In order to narrow down the candidate drugs predicted by the deep neural network framework based on the identified biomarkers, we designed two filters considering drug regulation ability and drug sensitivity. With the help of the CMap dataset, we could know whether a gene was upregulated or downregulated after treating the small molecule compound. The abnormal up or down gene expression could be found by comparing the gene expression of identified biomarkers to the later skin stage. The goal for the first filter is to select candidate drugs, which could reverse the abnormal gene expression. Afterwards, we used the drug sensitivity dataset (PRISM Repurposing Primary Screen) to consider drug sensitivity. The second filter aims to find the drugs with around zero values implying that they would not influence the cell line too much since we are not going to kill or proliferate cells toward the skin corresponding cell line. Consequently, we proposed niridazole, liothyroninr, decitabine, pinacidil, and allantoin as a multiple-molecule drug for mitigating skin aging from young adulthood to middle age; and allantoin, diclofenac, mepyramime, resveratrol, and azathioprine as multiple-molecule drug for mitigating skin aging from middle age to old age. The drug targets with their corresponding drugs are shown in Tables S4 and S5.

## 3. Discussion

### 3.1. Investigating Skin Aging Molecular Progression Mechanisms by Differential Core Signaling Pathways from Young-Adult to Middle-Aged Human Skin Aging

In the first core pathway of young-adult skin aging only as shown in Figure 4, microenvironment factor fatty acid synthase FASN can promote cell proliferation, DNA repair, and cell-cycle arrest and interact with receptor ESR1 via crucial signaling transduction proteins PRR4, LMNA, GOT1, and CHEK2 to regulate TFs SIRT6, PARP1, and JUN. The signaling protein LMNA, which is an endogenous activator of TF SIRT6, could promote SIRT6-mediated downstream functions upon DNA damage. Moreover, protein LMNA could directly bind and activate TF SIRT6 toward histone deacetylation [36]. The TF SIRT6, which could control the longevity and regulation of DNA repair, could promote DNA repair and cell proliferation through the downregulation of the target gene *RBBP8*, which was mediated by deacetylation [37]. TF SIRT6 could also promote DNA repair under oxidative stress by activating TF PARP1 to upregulate target gene *XRCC1* [38]. TF PARP1 serves as a genomic caretaker by participating in several molecular mechanisms such as DNA repair and cell-cycle regulation. Therefore, PARP1 was considered as a longevity assurance and aging-promoting factor [39]. The target gene *XRCC1* upregulated by PARP1 was required for the viable and efficient repair for DNA single-strand breaks [40]. The TF JUN was also activated by the signaling transduction proteins GOT1 and CHEK2. The signaling transduction protein CHEK2 initiated by oxidative stress could regulate target gene *BRCA2* and *KPNA2* through interacting with TF JUN. In human cell, the serine kinase CHEK2 could induce the appropriate cellular response such as cell cycle checkpoint activation and DNA repair depending on the extent of damage, the cell type, and other factor. CHEK2 could participate in DNA repair by phosphorylating the target gene *BRCA2* through TF JUN [41]. The karyopherin alpha2 *KPNA2* expression had been reported to be induced in various proliferative skin disorders such as psoriasis and squamous cell carcinoma [42]. When the target gene *KPNA2* was downregulated by TF JUN, cell proliferation, cell cycle and DNA repair induced by CHEK2 were indirectly promoted.

In the core pathways of both young-adult and middle-aged skin aging, the microenvironment factor FASLG binds receptor FAS to regulate TF E2F7 through signaling transduction proteins DAXX, MAP3K5 and AIFM1. Responding to ROS, the microenvironment factor FASLG was activated, then binding to death receptor FAS to promote apoptosis pathway [43]. Signaling transduction protein MAP3K5 known as apoptosis signal-regulating kinase 1 (ASK1) could respond to oxidative stress and be activated [44]. Target gene *APAF1* is the core of the apoptosome, was activated by TF E2F7 to trigger the mitochondrial apoptotic pathway. Furthermore, target gene *APAF1* was also involved in the maintenance of genomic stability by the cell-cycle arrest response elicited upon DNA damage and promoted apoptosis [45]. For other pathways in both young-adult and middle-aged skin aging, microenvironment factor CXCL1 bound the G-protein coupled receptor CXCR2 to activate signaling transduction protein CDKN1A through protein CENPJ. Protein CDKN1A known as CIP1, was a potent cyclin-dependent kinase inhibitor to regulate TFs E2F7, JUN, FOXM1, and miRNA MIR26B. First, protein CDKN1A transmits signal to AIFM1 so as to activate TF E2F7 to enhance apoptosis and cell-cycle arrest. TF JUN was also activated by protein CDNK1A to regulate FOXM1. Then TF FOXM1 upregulated target gene *CAT*, which was known as ROS detoxification enzyme and could defend the ROS accumulation [46]. After miRNA MIR26B inhibited the target gene *KPNA2*, the cell proliferation could be promoted [47].

In core pathways of middle-aged skin aging only, microenvironment factor CXCL1 also could regulate TF FOXM1 through signaling transduction proteins CENNPJ, CDKN1A, CDK4, and DCDC2 to trigger target gene *CCNB1* as shown in Figure 4. Cyclin dependent kinase 4 CDK4, which was modified by phosphorylation, is a positive regulator of cell cycle entry and can stabilize and activate FOXM1, thereby promote cell cycle and suppress the levels of reactive oxygen species [48]. TF FOXM1 also had been reported to be essential for proper cell cycle progression via activating cell cycle gene *CCNB1* for propelling specific cell cycle phase and inhibition ROS accumulation [49]. For another pathway, the microenvironment factor IGF1 was received by receptor IGF1R to activate FOXO3 via signaling transduction proteins HMGCS2, ARRB1, PDK1 and AKT1. The protein AKT1, which was modified by phosphorylation, could activate TF FOXO3. Protein phosphoinositide-dependent kinase PDK1 was one of the upstream kinases that activate AKT1. After AKT1, which is a key regulator of the PI3K/AKT1 signaling cascade controlling cell growth and survival, was activated and modified by phosphorylation, TF FOXO3 would be activated [50]. Moreover, it had been reported that the enhanced ROS production might further activate the signal of PI3K/AKT pathway, thus establishing a self-perpetuating cycle leading to further aging [51]. TF FOXO3, which was modified by phosphorylation, could downregulate target genes *SESN3* and *GADD45A* [52]. TF FOXO3 could decline ROS rescue pathway through downregulating the peroxiredoxin gene *SESN3*, which is responsible for the biphasic ROS accumulation. Therefore, FOXO3-induced ROS was increased and then accelerated for apoptosis and DNA damage [53]. Furthermore, phosphorylated FOXO3 also inhibited proapoptotic activity such as cell-cycle arrest by downregulating *GADD45A* [54]. The cause of the pleiotropic action of *GADD45* members, a decreased inducibility, might lead to far-reaching consequences such as DNA damage accumulation and disorder of cellular homeostasis and could eventually contribute to the aging process [55]. Therefore, we suggest that the downregulation of *GADD45A* also promotes ROS accumulation through cell-cycle arrest and the inhibition of proapoptotic activity.

### 3.2. Investigating Skin Aging Molecular Progression Mechanisms by Differential Core Signaling Pathways from Middle-Aged to Elderly Human Skin Aging

According to the core pathways of middle-aged skin aging only in Figure 5, the ligand TNF can inhibit collagen stability and skin homeostasis through receptor TNFRSF1A by transmitting the signal through significant signaling transduction proteins GABPA and STAT1 to TF GATA2. The proinflammatory cytokine tumor necrosis factor-alpha (TNF-A) inhibits collagen synthesis and enhances collagen degradation via increasing the production of target gene *MMP9*. It also increases the risk of cutaneous infections in the elderly by reducing skin immunity [56]. The activation of STAT1 is modified by phosphorylation. STAT1 has also been indicated as a potential target in the treatment of psoriasis, which is a chronic skin diseases [57]. TF GATA2 could upregulate target gene *MMP9* to digest collagen type IV, which is an important component of the basement membrane in skin [58].

For the next pathway, ligand NGF can promote skin homeostasis through receptor NTRK1 to transmit the significant signal via signaling transduction proteins EME1, HSPB1, NEDD9, and CPNE2 to upregulate TF ETS1. In human skin, proliferating keratinocytes release NGF in an increasing amount. Receptor NTRK1, known as tyrosine kinase receptor (TrkA) is the high-affinity receptor for NGF. At the skin level, NTRK1 could mediate NGF-induced keratinocyte proliferation [59]. Note that protein NEDD9 could be modified by phosphorylation in human skin [60]. TF ETS1, which was regulated through the signaling pathway activated by ligand NGF, has been identified to be associate with skin aging [60]. The expression levels of MIR-573 were found to be lower in melanoma tissues and cell lines when compared to normal skin tissue. Moreover, MIR-573 reduction was demonstrated to be essential in melanoma initiation and progression [61]. Target gene *ERRFI1*, which was modified by hypermethylation, is required for proper epidermal homeostasis [62,63].

Focusing on core pathways in both middle-aged and elderly skin aging in Figure 5, the ligand NGF inhibits apoptosis and promotes cell-cycle arrest when received by receptor NTRK1 to activate TF GATA2 via signaling transduction proteins KPNA2, KAT5, CST2, and HRAS. Protein KAT5, which was modified by phosphorylation, has been presumed to serve as a potential biomarker for melanoma therapeutic target [64]. NGF can not only rescue human epidermal keratinocytes from spontaneous and UVB-induced apoptosis via NTRK1, but also protect keratinocytes from cell death via target gene *BCL2* family of apoptosis inhibitors [59]. Antiapoptotic function of target gene *BCL2* is regulated by phosphorylation. In addition, target gene *BCL2* could not only regulate cell cycle progression, but also act as an antioxidant that may regulate intracellular ROS. Expression of target gene *BCL2* has been observed to increase upon the induction of a senescence-like growth arrest or apoptosis by oxidative stress [65,66].

In the next core pathway of both middle-aged and elderly skin aging, the ligand KITLG promotes melanin synthesis, DNA damage, and inhibits cell-cycle arrest by modulating TFs AR and MITF via signaling transduction proteins FAM83H, HSPB1, PAX3, ATF5, and H2AFB2. The tyrosine kinase receptor KIT, its ligand KITLG, and TF MITF have been reported to play an important role of initiating and regulating signaling systems and transcription factors of melanin production. TF MITF also regulates melanocyte pigmentation by inducing target gene *TYR* [67]. Moreover, a previous study supposed that PAX3 and SOX10 could act together to induce the expression of MITF [68]. Target gene *CDH1*, which is downregulated by TF AR, has been reported to be regulated by phosphorylation [69]. It has been reported that cells lacking target gene *CDH1* have a shortened G1 phase, accumulate DNA damage, and undergo apoptosis [70].

In the final core pathways of both middle-aged and elderly skin aging, the ligand CYR61 could modify skin homeostasis and melanin synthesis through receptor LRP1 to transmit signal by signaling transduction proteins RAMP1, MCM2, GEMIN4 and NOP56 for upregulating TF ETS1. Responding to oxidative stress, CYR61 was elevated in the dermis of chronologically aged human skin, promoting aberrant collagen homeostasis by downregulating collagen members, the major structural protein in skin, to promote collagen degradation [71,72]. The loss of target gene *COL17A1* and MCM2 expression in advanced aged skin has been found to eventually cause epidermal thinning [73].

Focusing on the first core pathway of elderly skin aging only in Figure 5, through the signaling transduction starting from the ligand CYR61, TF NDUFS4 can promote apoptosis and DNA damage through signaling transduction proteins CPNE2, MYH9 and ERRC6. The ligand CYR61 interacting with receptor LRP1 has also been indicated to contribute to CCN1-induced ROS accumulation and CCN1/TNFA-induced apoptosis [74]. With the downregulating target gene *CASP3* by TF NDUFS4, senescence fibroblast can resist apoptosis death [75].

In the final core pathway of elderly skin aging only, the ligand IL6 could be accepted by receptor IL6R and then the significant signal is transmitted through signaling transduction proteins RHOB and CDK20 to activate TF YAP1. Proinflammatory cytokine IL6 has been suggested to be a biomarker of health status in the elderly [76]. TF YAP1 has been identified to play a physiological role in skin homeostasis, which can promote cell proliferation in the basal layer [77]. Knockdown of target gene *CDC5L* induces mitotic arrest and DNA damage [78].

### 3.3. The Genetic and Epigenetic Molecular Progression Mechanisms from Young-Adult to Elderly Human Skin Aging

The overview of overall skin aging molecular progression mechanisms is shown in Figure 6. Microenvironments trigger corresponding ligand signals to initiate some cellular dysfunctions affecting skin aging progression. Thus, core signaling pathways with the genetic and epigenetic modifications play a significant role in cellular dysfunctions of signaling transductions for each stage of skin aging.

In Figure 6, the core pathways of young-adult skin aging only, ligand FASN (oxidative stress) binds to receptor ESR1 to mediate two pathways. Responding to DNA damage signal to cause of oxidative stress, LMNA directly binds and activates TF SIRT6 toward histone deacetylation [36]. Activated SIRT6 promotes DNA repair cell-cycle and proliferation through the downregulating gene *RBBP8*, which was modified by deacetylation [37]. In addition, TF SIRT6 also promotes DNA repair and cell cycle under oxidative stress by activating TF PARP1 to upregulate target gene *XRCC1* [38]. Transduction protein CHEK2 also responds to oxidative stress from ligand FASN to activate TF JUN. TF JUN promotes DNA repair through phosphorylating target gene *BRCA2* [41]. Moreover, TF JUN could downregulate target gene *KPNA2* to promote cell proliferation, cell cycle and DNA repair [42].

In the core pathways of both young-adult and middle-aged skin aging, ligand CXCL1 (oxidative stress) binds to receptor CXCR2 to trigger protein CDKN1A. Activated protein CDKN1A not only upregulates target gene *CAT* to defend ROS accumulation through TF JUN and FOXM1, but also inhibits target gene *KPNA2* to promote cell proliferation by activating MIR26B [46,47]. Next, responding to ROS induced from DNA damage, the ligand FASLG interacts with FAS to initiate apoptosis pathway. Signaling transduction protein MAP3K5, activated by oxidative stress, triggers TF E2F7 to downregulate target gene *APAF1* and thereby involve in the maintenance of cell-cycle arrest upon DNA damage and promoting apoptosis [43,44,45]. Hence, in order to fight to the excessive accumulation of ROS upon decreasing the ability of DNA repair from young adulthood to middle-age, functions of apoptosis and cell-cycle arrest are raised.

In the core pathways of middle-aged skin aging only, the ligand CXCL1 (oxidative response) interacts with receptor CXCR2 and also activates signaling transduction protein CDK4. Phosphorylation of CDK4 positively regulates cell cycle entry and can stabilize and activate FOXM1 to upregulate target gene *CCNB1* to promote cell cycle phase and suppress the level of ROS [48,49]. Moreover, the ligand IGF1 (oxidative response) is received by receptor IGF1R to activate PI3K/AKT signaling pathway. Transduction protein AKT1 can activate TF FOXO3 through the modification by phosphorylation. Furthermore, TF FOXO3, which is modified by phosphorylation, downregulates genes *SESN3* and *GADD45A*. With the silence of target gene *SESN3*, ROS rescue pathway is declined, thus accelerating apoptosis with the increment of FOXO3-induced ROS. In addition, TF FOXO3 downregulates target gene *GADD45A* to promote ROS accumulation through cell-cycle arrest. The ligand TNF (proinflammatory cytokine) can inhibit collagen stability and skin homeostasis through activating TNFRSF1A and initiate the corresponding pathway. TF GATA2 was activated by phosphorylated transduction protein STAT1 to upregulate target gene *MMP9*. Increased gene *MMP9* can inhibit collagen synthesis and enhance collagen degradation. The ligand NGF (proliferating keratinocytes) interacts with receptor NTRK1 to activate TF ETS1, which was identified to be associative with skin aging [59,60]. MIR573 is activated by TF ETS1 and then negatively regulates gene *ERRFI1*. Downregulated gene *ERRFI1*, which was modified by hypermethylation, can maintain proper epidermal homeostasis [62,63].

In the core pathways of both middle-aged and elderly skin aging, the ligand NGF/NTRK1 (signal of positive regulation of Ras signaling pathway) activates TF GATA2 so as to regulate gene *BCL2* to protect keratinocytes from cell death [59]. Target gene *BCL2* not only triggers antiapoptotic function through the modification by phosphorylation, but also regulates cell cycle progression to act as an antioxidant of intracellular ROS [65,66]. The ligand KITLG interacts with receptor KIT to promote melanin synthesis, DNA damage, and inhibits cell-cycle arrest by activating TF MITF through signaling transduction. TF MITF regulates melanocyte pigmentation by inducing gene *TYR* [67]. Due to the downregulation of gene *CDH1*, which is modified by phosphorylation, by TF AR, G1 phase is shortened to accumulate DNA damage and undergo apoptosis [69,70]. Next, the ligand CYR61 (oxidative stress) is received by receptor LRP1 to downregulate collagen members and promote collagen degradation [71,72]. After TF ETS1 is activated by signaling transduction, target gene *COL17A1* can be downregulated to destroy the balance between collagen stability and skin homeostasis and eventually cause epidermal thinning [73].

In the core pathways of elderly skin aging only, the ligand CYR61 (oxidative stress) interacts with receptor LRP1 so as to contribute to the CCN1-induced ROS accumulation. When target gene *CASP3* is downregulated by TF NDUFS4, senescence fibroblast could resist apoptosis death [74,75]. Moreover, since the ligand IL6 interacts with receptor IL6R to activate TF YAP1, which could promote cell proliferation in basal layer, it has been identified to play a physiological role in skin homeostasis [77]. Note that IL6 has been suggested as a biomarker of elderly health status [76]. After TF YAP1 downregulating target gene *CDC5L* through activating MIR126, functions of mitotic arrest and DNA damage were activated [78].

### 3.4. Two Multiple-Molecule Drugs Based on Identified Biomarkers to Mitigate Human Skin Aging

For mitigating the skin aging from young adulthood to middle age, we proposed a multiple-molecule drug including niridazole, liothyronine, decitabine, pinacidil, and allantoin. The drug targets were AIFM1, CAT, IGF1R, and LMNA as shown in Table 2. The black dot in Table 2 represents the proposed small molecules target to which identified biomarker (drug target). For instance, the niridazole has more potential to target to AIFM1 and CAT. Niridazole, an antiparasitic drug, could suppress delayed dermal hypersensitivity [79]. Studies have shown that combined therapy with liothyronine improved the treatment of hypothyroidism [80,81]. Decitabine, a DNA methyltransferase, induced changes in gene expression and cellular behavior associated with a regenerative response. Furthermore, wounds treated by decitabine were able to participate in regeneration [82]. Pinacidil is an effective antihypertensive drug for the treatment of mild to moderate essential hypertension [83]. In the meanwhile, according to the findings of one study, pinacidil may be utilized to prevent from UV-induced skin aging [84]. It is noted that allantoin, which is found in plants like chamomile, wheat sprouts, sugar beet, and comfrey, has been widely used in anti-aging serum [85,86]. Allantoin is also a well-known anti-irritating and hydrating agent as well as a peeling agent for skin [87,88].

For mitigating the skin aging from middle-aged to elderly, we proposed a multiple-molecule drug consisting of allantoin, diclofenac, mepyramine, resveratrol, and azathioprine. The drug targets were MMP9, IL6, BCL2, and CASP3 as shown in Table 3. In Table 3, the black dot shows the drug target to each specific small molecule. For example, the drug target for allantoin are MMP9 and IL6. Diclofenac is a nonsteroidal anti-inflammatory drug. It has been used to treat actinic keratoses developing in fair-skinned individuals with a history of overexposure to ultraviolet light [89]. To mepyramine, it works by preventing the action of histamine, which is a compound produced by the body when getting venom from insect bites [90]. Moreover, one study mentioned the stimulation from histamine would upregulate matrix metalloproteinase 9 (MMP9), which is also our proposed drug target for mepyramine [91]. Resveratrol is abundant in grape skin and seeds [92]. Responding to infection, stress, injury, bacteria or fungal infections, and UV-irradiation, it a popular ingredient in skincare products [93]. In the field of dermatology, azathioprine is an effective immunosuppressant that is extremely valuable in treating pemphigoid, generalized eczematous disorders, and actinic dermatitis [94]. Taken together, most of the proposed small-molecule compounds are approved by the U.S. Food and Drug Administration (FDA). Drug repurposing for identifying new uses of old drugs with the proposed systems biology approaches might provide the alternative way to find the effective drugs for mitigating skin aging.

### 3.5. The Limitations and Advantages to the Proposed Systems Medicine Design Procedure for Human Skin Aging

Gene expression has been widely used to infer other molecular type measures, such as proteomics, copy number variation, and mutation. In this study, we used human skin microarray data processed with cubic spline interpolation to help us construct GWGENs by system identification method via solving constrained linear least-squares estimation problem. After that, we computed Akaike’s information criterion (AIC) for each gene to prune false positives. Increasing samples through data interpolation and computing AIC for detecting real systems order, we conquered the overfitting issue. Even though we applied AIC and performed the data interpolation for increasing sample size in each skin aging stage, it is noted that the estimated real GWGENs are near-optimum solutions but not unique solutions. Furthermore, we include basal level in protein, gene, miRNA, and lncRNA systems modeling. These terms imply the unknown interaction or epigenetic modification, and mutation. If we found a basal level change, which was higher than a threshold, we inferred the corresponding node was influenced by epigenetic modification or mutation. These findings have to be verified by a literature survey. Based on the progression molecular mechanisms in each skin aging stage, we could identify essential biomarkers. For exploring the drug–target interaction to our identified biomarkers, we trained a deep neural network of drug–target interaction in advance. In the drug–target data which we used to train the prediction model, if pairs have not been mentioned as known interactions in the BindingDB, we would assign them in the group of negative samples, meaning no interaction. However, the negative samples in our study do not mean without interaction. They might just be lack of experimental evidence or record nowadays. Although the proposed system medicine design procedure exists the aforementioned limitations, it still provides another viewpoint to shed the light on the human skin aging progression based on system level. Moreover, drug repurposing strategy, giving new uses for old drugs, has been used in this study. Most of the suggested small molecules are approved by the FDA, which could shorten the time of clinical trials. Integrating systems biology approaches, deep learning framework and the design of two filters, we not only transferred biological knowledge into engineering interpretation but also applied them to drug discovery efficiently.

## 4. Materials and Methods

### 4.1. Overview of Systems Medicine Design Procedure of Human Skin Aging

In order to further understand skin aging molecular mechanisms from young adulthood to old age, we proposed a research flowchart as shown in Figure 2. At first, we collect several regulation and interaction databases including DIP [95], IntAct [96], BioGRID [97], BIND [98], MINT [99], HTRIdb [100], ITFP [101], Transfac [102], CircuitDB2 [103], and TargetScan [104] to construct the candidate GWGEN, which is composed of candidate protein-protein interaction network (PPIN) and candidate gene regulatory network (GRN). Moreover, the candidate GWGEN is a Boolean matrix. If two nodes have interaction, we would give one; if two nodes do not have interaction, we would give zero in it. With three-stage preprocessed microarray data, we then identify real GWGENs by system identification method and system order detection scheme. Since real GWGENs are still too complicated to investigate the skin aging progression mechanisms, we apply principal network projection (PNP) method to extract core GWGENs from real GWGENs based on the projection values. Subsequently, we denote the core signaling pathways in the style of KEGG pathways. According to the core signaling pathways, we investigate skin aging molecular mechanisms and identify essential biomarkers for young adulthood to middle age and middle age to old age, respectively. After that, we used the trained deep neural network of drug-target interaction to predict potential candidate drugs, which hold higher probability to have interactions with identified biomarkers. To narrow down the candidate drugs, we design two filters considering drug regulation ability and drug sensitivity by CMap [34] and PRISM Repurposing dataset [105]. Consequently, we propose two multiple-molecule drugs for slowing down human skin aging from young adulthood to middle age and from middle age to old age, respectively.

### 4.2. Data Preprocessing of Human Skin Microarray Data

We obtained human skin microarray data from GSE18876 containing the gene expression level of male skin. It included 50 ages in the range from 19 to 86 years old with 29,226 probes. One study has shown that *OR52N2*, *SIRT6*, *CPT1B*, *TUBAL3*, *COL1A1* and *MATN4* were significantly regulated with age. Furthermore, it also indicated that gene expressions of *OR52N2*, *SIRT6* and *CPT1B* increased with age and gene expressions of *TUBAL3*, *COL1A1* and *MATN4* decreased with age [106]. Therefore, we sketched the changes of gene expression levels of these typical genes. Based on this line graph and gene expression trend in aforementioned study, we defined young-adult, middle-aged and elderly skin as 19 to 45 years old, 43 to 65 years old and 64 to 86 years old, respectively. That is, the averages of gene expressions of *OR52N2*, *SIRT6* and *CPT1B* increased and the averages of gene expressions of *TUBAL3*, *COL1A1* and *MATN4* decreased from young adult stage to middle age, and then to old age in human male skin. In the estimation problem, one would easily face overfitting issue when the sample size is small and the feature size is big [107]. Hence, in this study, firstly, we increased the sample size to 500 for each skin aging stage by performing cubic spline data interpolation via *splin*, a MATLAB function [108,109,110]. Secondly, we utilized system order detection scheme by computing the AIC value to prune the false positives in the candidate GWGEN for finding the real GWGENs of the human skin aging systems. The more details would be discussed in the Section 4.5.

### 4.3. Dynamic Systems Modeling for the Candidate GWGEN

The candidate GWGWN consisting of PPIN and GRN. It is noted that GRN also includes miRNA regulation network and lncRNA regulation network. In the following contents, we would take PPIN and GRN as an example, and the rest of them could be found in Supplementary Materials. The PPIs of human-protein *i* in the candidate PPIN can be described as a dynamic equation shown as below:

(1) $$\begin{array}{ll} p_{i}(t+1) & =p_{i}(t)+\sum_{j=1}^{I_{i}}\alpha_{ij}^{P}p_{i}(t)p_{j}(t)-\sigma_{i}^{P}p_{i}(t)+\lambda_{i}^{P}g_{i}(t)+\beta_{i}^{P}+\epsilon_{i}^{P}(t)\\ & ,\;\;\mathrm{for}\;\;i=1,\ldots ,I,\;-\sigma_{i}^{P}\leq 0\;\mathrm{and}\;\lambda_{i}^{P}\geq 0. \end{array}$$

where $p_{i}(t),\;p_{j}(t),$ and $g_{i}(t)$ indicate the expression levels of the *i*th protein, the *j*th protein, and the *i*th gene at time t, respectively; $\alpha_{ij}$ denotes the interactive abilities between the *i*th protein with the *j*th protein in human skin cells; $\sigma_{i}^{P}$ represents the degradation rate of the *i*th protein; $\lambda_{i}^{P}$ indicates the translation effect from the corresponding mRNA to the *i*th protein; The basal level $\beta_{i}^{P}$ signifies the regulations from other unknown regulators to the *i*th protein; $I_{i}$ denotes the number of human proteins interacting with the ith protein in the candidate GWGENs; $\epsilon_{i}^{P}(t)$ signifies the noise of the *i*th protein owing to model uncertainty or measurement noise at time *t*.

The *k* gene in the candidate GRN can be represented as a dynamic equation in the following:

(2) $$\begin{array}{ll} g_{k}(t+1)= & g_{k}(t)+\sum_{i=1}^{I_{k}}a_{ki}^{G}p_{i}(t)-\sum_{r=1}^{R_{k}}b_{kr}^{G}g_{k}(t)m_{r}(t)+\sum_{\ell =1}^{L_{k}}c_{k\ell }^{G}o_{\ell }(t)-\mu_{k}^{G}g_{k}(t)\\ & +\delta_{k}^{G}+\omega_{k}^{G}(t)\;\mathrm{for}\;\;k=1,2,\ldots ,K,\;-b_{kr}^{G}\leq 0\;\;\mathrm{and}\;\;-\mu_{k}^{G}\leq 0 \end{array}$$

where $g_{k}(t)$, $p_{i}(t)$, $m_{r}(t)$, and $o_{\ell }(t)$ indicate the expression level of the *k*th gene, the *i*th transcription factor(TF), the *r*th miRNA and the ℓth lncRNA at time *t*, respectively; $a_{ki}^{G}$, $-b_{kr}^{G}$, and $c_{k\ell }^{G}$ represent the regulatory abilities of the *i*th TF, the repression ability of the *r*th miRNA, and the regulatory ability of the ℓth lncRNA on the *k*th gene, respectively; $-\mu_{k}^{G}$ signifies the degradation rate of the gene expression of the *k*th gene; The basal level $\delta_{k}^{G}$ denotes the regulations from other unknown regulators to the *k*th gene such as phosphorylation; $\omega_{k}^{G}(t)$ signifies the noise of the *k*th gene owing to model uncertainty or measurement noise at time *t*; $I_{k}$, $R_{k}$, and $L_{k}$ mean the total number of TFs, miRNAs, and lncRNAs in the candidate GRN, respectively. Note that the biological regulatory mechanisms in skin cell in (2) involve TF transcription regulations by $\sum_{i=1}^{I_{k}}a_{ki}^{G}p_{i}(t)$, miRNA repressions by $-\sum_{r=1}^{R_{k}}b_{kr}^{G}g_{k}(t)m_{r}(t)$, lncRNA regulation by $\sum_{\ell =1}^{L_{k}}c_{k\ell }^{G}o_{\ell }(t)$, the mRNA degradation by $-\mu_{k}^{G}g_{k}(t)$, the basal level by $\delta_{k}^{G}$, and the noise by $\omega_{k}^{G}(t)$. In this study, the effect of post-translational modification on skin aging is considered by the basal level term $\delta_{k}^{G}$.

### 4.4. Systems Identification Approach in the Candidate GWGEN via Microarray Data

After systems modeling by Equations (1)–(4), we then perform the systems identification by solving the parameter estimation problems. The PPIN in Equation (1) can be rewritten in the following linear regression form:

(3) $$\begin{array}{ll} p_{i}(t+1) & =\left[\begin{array}{cccccc} p_{1}(t)p_{i}(t) & \cdots & p_{I_{i}}(t)p_{i}(t) & g_{i}(t) & p_{i}(t) & 1 \end{array}\right]\left[\begin{array}{c} \alpha_{i1}^{P}\\ \vdots \\ \alpha_{iI_{i}}^{P}\\ \lambda_{i}^{P}\\ 1-\sigma_{i}^{P}\\ \beta_{i}^{P} \end{array}\right]+\epsilon_{i}^{P}(t)\\ & =\psi_{i}^{P}(t)\theta_{i}^{P}+\epsilon_{i}^{P}(t)\;\;for\;\;i=1,2,\ldots ,I. \end{array}$$

where $\psi_{i}^{P}(t),$ represents the regression vector that can be obtained from the microarray data and $\theta_{i}^{P}$ denotes the unknown parameter vector to be estimated for the *i*th protein in PPIN.

Furthermore, the Equation (3) of the *i*th protein can be augmented for $Y_{i}$ time points shown as below:

(4) $$\left[\begin{array}{c} p_{i}(t_{2})\\ p_{i}(t_{3})\\ \vdots \\ p_{i}(t_{Y_{i}+1}) \end{array}\right]=\left[\begin{array}{c} \psi_{i}^{P}(t_{1})\\ \psi_{i}^{P}(t_{2})\\ \vdots \\ \psi_{i}^{P}(t_{Y_{i}}) \end{array}\right]\theta_{i}^{P}+\left[\begin{array}{c} \epsilon_{i}^{P}(t_{1})\\ \epsilon_{i}^{P}(t_{2})\\ \vdots \\ \epsilon_{i}^{P}(t_{Y_{i}}) \end{array}\right],\;for\;\;i=1,2,\ldots ,I,$$

which can also be simplified by

(5) $$P_{i}=\Psi_{i}^{P}\theta_{i}^{P}+E_{i}^{P},\;for\;\;i=1,2,\ldots ,I$$

where

$$P_{i}=\left[\begin{array}{c} p_{i}(t_{2})\\ p_{i}(t_{3})\\ \vdots \\ p_{i}(t_{Y_{i}+1}) \end{array}\right],\;\Psi_{i}^{P}=\left[\begin{array}{c} \psi_{i}^{P}(t_{1})\\ \psi_{i}^{P}(t_{2})\\ \vdots \\ \psi_{i}^{P}(t_{Y_{i}}) \end{array}\right],\;E_{i}^{P}=\left[\begin{array}{c} \epsilon_{i}^{P}(t_{1})\\ \epsilon_{i}^{P}(t_{2})\\ \vdots \\ \epsilon_{i}^{P}(t_{Y_{i}}) \end{array}\right].$$

Therefore, the interaction parameters in the vector $\theta_{i}^{P}$ can be estimated by solving the following constrained least-squares estimation problem:

(6) $${\hat{\theta }}_{i}^{P}=\underset{\theta_{i}^{P}}{\min}\frac{1}{2}{\left\|\Psi_{i}^{P}\theta_{i}^{P}-P_{i}\right\|}_{2}^{2},\;\;\mathrm{subject}\;\mathrm{to}\;\;A^{P}\theta_{i}^{P}\leq b^{P},$$

where

$$A^{P}=\left[\begin{array}{cccccc} 0 & \cdots & 0 & -1 & 0 & 0\\ 0 & \cdots & 0 & 0 & 1 & 0 \end{array}\right]\in {\mathbb{R}}^{2\times (I_{k}+3)},\;b^{P}=\left[\begin{array}{c} 0\\ 1 \end{array}\right].$$

To estimate the interaction parameters in (1) by solving the parameter estimation problem in (6), we use an optimization toolbox function *lsqlin* in MATLAB. Simultaneously, we ensure the protein translation rate $\lambda_{i}^{P}$ and the protein degradation rate $-\sigma_{i}^{P}$ to always be non-negative and non-positive value, respectively; that is, $\lambda_{i}^{P}\geq 0$ and $-\sigma_{i}^{P}\leq 0$.

Similarly, we rewrite the dynamic equation of GRN in the Equation (2) as the following linear regression form:

(7) $$\begin{array}{lll} g_{k}(t+1) & =[p_{1}(t)\;\cdots \;p_{I_{k}}(t)\;g_{k}(t)m_{1}(t)\;\cdots \;g_{k}(t)m_{R_{k}}(t) & o_{1}(t)\;\cdots \;o_{L_{k}}(t)\;g(t)\;1\;]\left[\begin{array}{c} a_{k_{1}}^{G}\\ \vdots \\ a_{kI_{k}}^{G}\\ -b_{k_{1}}^{G}\\ \vdots \\ -b_{kR_{k}}^{G}\\ c_{k_{1}}^{G}\\ \vdots \\ c_{kL_{k}}^{G}\\ 1-\mu_{k}^{G}\\ \delta_{k}^{G} \end{array}\right]\\ & +\omega_{k}^{G}(t)=\psi_{k}^{G}(t)\theta_{k}^{G}+\omega_{k}^{G}(t),\;\;for\;\;k=1,2,\ldots ,K & \end{array}$$

where $\psi_{k}^{G}(t),$ represents the regression vector that can be obtained from the microarray data and $\theta_{k}^{G}$ signifies the unknown parameter vector estimated for the *k*th gene in the GRN. Moreover, Equation (7) can be augmented for $Y_{k}$ time points in the following form:

(8) $$\left[\begin{array}{c} g_{k}(t_{2})\\ g_{k}(t_{3})\\ \vdots \\ g_{k}(t_{Y_{k}+1}) \end{array}\right]=\left[\begin{array}{c} \psi_{k}^{G}(t_{1})\\ \psi_{k}^{G}(t_{2})\\ \vdots \\ \psi_{k}^{G}(t_{Y_{k}}) \end{array}\right]\theta_{k}^{G}+\left[\begin{array}{c} \omega_{k}^{G}(t_{1})\\ \omega_{k}^{G}(t_{2})\\ \vdots \\ \omega_{k}^{G}(t_{Y_{k}}) \end{array}\right],\;\;\mathrm{for}\;\;k=1,2,\ldots ,K$$

Next, we simplify the Equation (8) as below:

(9) $$G_{k}=\Psi_{k}^{G}\theta_{k}^{G}+\Omega_{k}^{G},\;\mathrm{for}\;k=1,2,\ldots ,K$$

where

$$G_{k}=\left[\begin{array}{c} g_{k}(t_{2})\\ g_{k}(t_{3})\\ \vdots \\ g_{k}(t_{Y_{k}+1}) \end{array}\right],\;\Psi_{k}^{G}=\left[\begin{array}{c} \psi_{k}^{G}(t_{1})\\ \psi_{k}^{G}(t_{2})\\ \vdots \\ \psi_{k}^{G}(t_{Y_{k}}) \end{array}\right],\;\Omega_{k}^{G}=\left[\begin{array}{c} \omega_{k}^{G}(t_{1})\\ \omega_{k}^{G}(t_{2})\\ \vdots \\ \omega_{k}^{G}(t_{Y_{k}}) \end{array}\right].$$

Hence, the regulatory parameters in the vector $\theta_{k}^{G}$ can be estimated by solving the following constrained least-squares estimation problem:

(10) $${\hat{\theta }}_{k}^{G}=\underset{\theta_{k}^{G}}{\min}\frac{1}{2}{\left\|\Psi_{k}^{G}\theta_{k}^{G}-G_{k}\right\|}_{2}^{2},\;\;subject\;to\;\;A^{G}\theta_{k}^{G}\leq b^{G}\;$$

where

$$A^{G}=\left[\begin{array}{cccccccccccccc} 0 & 0 & \cdots & 0 & 1 & 0 & \cdots & 0 & 0 & 0 & \cdots & 0 & 0 & 0\\ 0 & 0 & \cdots & 0 & 0 & 1 & \cdots & 0 & 0 & 0 & \cdots & 0 & 0 & 0\\ \vdots & \vdots & \ddots & \vdots & \vdots & \vdots & \ddots & \vdots & \vdots & \vdots & \ddots & \vdots & \vdots & \vdots \\ 0 & 0 & \cdots & 0 & 0 & 0 & \cdots & 1 & 0 & 0 & \cdots & 0 & 0 & 0\\ 0 & 0 & \cdots & 0 & 0 & 0 & \cdots & 0 & 0 & 0 & \cdots & 0 & 1 & 0 \end{array}\right]\in {\mathbb{R}}^{\left(R_{k}+1)\times (I_{k}+R_{k}+L_{k}+2\right)}$$

$$,\;b^{G}=\left[\begin{array}{c} 0\\ \vdots \\ 1 \end{array}\right].$$

By applying the function *lsqlin* in MATLAB optimization toolbox to solve the parameter estimation problem in Equation (10), we can estimate the regulatory parameters for GRN equation in Equation (2). Furthermore, we ensure that the miRNA repression rate $-b_{kr}^{G}$ to be a nonpositive value and the gene degradation rate $-\mu_{k}^{G}$ to be a nonpositive value for k=1,2,…K and $r=1,2,\ldots R_{k}$.

### 4.5. Pruning False Positives in Candidate GWGENs to Obtain Real GWGENs by System Order Detection Scheme

Due to the collected data, which we used for constructing the candidate GWGEN, come from different databases, the various experimental conditions and noises might result in getting many false-positive interactions and regulations after doing system identification. Thus, we have to apply system order detection scheme by computing AIC to detect the real system order of PPI model in Equation (1) and GRN model in Equation (2). According to Akaike’s theory, the most accurate model has the smallest AIC value [111]. In other words, when the value of AIC achieves the minimum, the detected system order approaches to the real system order.

For PPI model in Equation (5), the AIC value of the *i*th protein can be defined in the following equation:

(11) $$AIC_{i}^{P}(K_{i})=\log\left\{\frac{1}{T_{i}}{\left[P_{i}-\Psi_{i}^{P}{\hat{\theta }}_{i}^{P}\right]}^{T}\left[P_{i}-\Psi_{i}^{P}{\hat{\theta }}_{i}^{P}\right]\right\}+\frac{2K_{i}}{T_{i}}$$

where ${\hat{\theta }}_{i}^{P}$ denotes the estimated interactive parameters of the *i*th protein from the solutions of the parameter estimation problem in Equation (6), and the covariance of estimated residual error is ${\left(\varsigma_{i}^{P}\right)}^{2}=\frac{1}{T_{i}}{\left[P_{i}-\Psi_{i}^{P}{\hat{\theta }}_{i}^{P}\right]}^{T}\left[P_{i}-\Psi_{i}^{P}{\hat{\theta }}_{i}^{P}\right]$. In order to find out the real system order $K_{i}^{*}$ of the *i*th protein in the PPI model so that $AIC_{i}^{P}(K_{i}^{*}),$ in Equation (11) can achieve the minimum value, we trade off the system order and the estimated residual error. By aforementioned system order detection method, PPIs with insignificant interaction abilities, which are out of $K_{i}^{*}$, could be regarded as false positives and be pruned away.

For the GRN model in Equation (9), AIC value of the *k*th gene can be defined as the following equation:

(12) $$AIC_{k}^{G}(I_{k},R_{k},L_{k})=\log\left\{\frac{1}{T_{k}}{\left[G_{k}-\Psi_{k}^{G}{\hat{\theta }}_{k}^{G}\right]}^{T}\left[G_{k}-\Psi_{k}^{G}{\hat{\theta }}_{k}^{G}\right]\right\}+\frac{\left(2I_{k}+R_{k}+L_{k}\right)}{T_{k}}$$

where ${\hat{\theta }}_{k}^{G}$ denotes the estimated regulatory parameters of the *k*th gene from the solutions of the parameter estimation problem in Equation (10), and the covariance of estimated residual error is ${\left(\varsigma_{k}^{G}\right)}^{2}=\frac{1}{T_{k}}{\left[G_{k}-\Psi_{k}^{G}{\hat{\theta }}_{k}^{G}\right]}^{T}\left[G_{k}-\Psi_{k}^{G}{\hat{\theta }}_{k}^{G}\right]$. In order to find out the real system order $I_{k}^{*}$, $R_{k}^{*}$, and $O_{k}^{*}$ of the *k*th gene in GRN so that $AIC_{k}^{G}(I^{*}{}_{k},R^{*}{}_{k},L^{*}{}_{k}),$ in (12) can achieve the minimum value, we trade off the system order and to estimate residual error. In this way, to *k*th gene, the gene regulations with insignificant regulatory abilities, which are out of $I_{k}^{*}$, $R_{k}^{*}$, and $O_{k}^{*}$, can be treated as false-positives and be pruned away from the candidate GRN. It is noted that we apply the same system order detection scheme on the miRNA model and the lncRNA model, which could be found in the Section S1.3 of Supplementary Materials.

After performing system identification and system order detection scheme, which pruned away the insignificant interactions and regulations in the candidate GWGEN, we eventually obtained the real GWGENs for three stage of human skin aging. However, it is still quite difficult to investigate the progression mechanisms of skin aging from these real GWGENs due to their high complexity. Here, we introduce the principal network projection (PNP) method to extract the core networks from the real GWGENs as core GWGENs to solve this issue. The details are described in the following section.

### 4.6. Extracting Core Networks from Real GWGENs by the Principal Network Projection Method

The PNP method is a network structure projection approach based on the principal singular values so as to reduce network dimension via deleting insignificant structures. In order to use the PNP method to extract the core networks from the real GWGENs, we have to construct a network matrix H consisting all of the estimated interactions and regulations in the real GWGEN (with the ith row denoting the interactions or regulations on the ith node, i.e., protein, gene, miRNA or lncRNA of real GWGEN) in the following formation:

(13) $$\begin{array}{ll} H= & \left[\begin{array}{ccccccccc} {\hat{\alpha }}_{11} & \cdots & {\hat{\alpha }}_{1I} & 0 & \cdots & 0 & 0 & \cdots & 0\\ \vdots & {\hat{\alpha }}_{ij} & \vdots & \vdots & 0 & \vdots & \vdots & 0 & \vdots \\ {\hat{\alpha }}_{I1} & \cdots & {\hat{\alpha }}_{II} & 0 & \cdots & 0 & 0 & \cdots & 0\\ {\hat{a}}_{11}^{G} & \cdots & {\hat{a}}_{1I}^{G} & -{\hat{b}}_{11}^{G} & \cdots & -{\hat{b}}_{1R}^{G} & {\hat{c}}_{11}^{G} & \cdots & {\hat{c}}_{1Z}^{G}\\ \vdots & {\hat{a}}_{ki}^{G} & \vdots & \vdots & -{\hat{b}}_{kr}^{G} & \vdots & \vdots & {\hat{c}}_{kz}^{G} & \vdots \\ {\hat{a}}_{K1}^{G} & \cdots & {\hat{a}}_{KI}^{G} & -{\hat{b}}_{K1}^{G} & \cdots & -{\hat{b}}_{KR}^{G} & {\hat{c}}_{K1}^{G} & \cdots & {\hat{c}}_{KZ}^{G}\\ {\hat{a}}_{11}^{M} & \cdots & {\hat{a}}_{1I}^{M} & -{\hat{b}}_{11}^{M} & \cdots & -{\hat{b}}_{1R}^{M} & {\hat{c}}_{11}^{M} & \cdots & {\hat{c}}_{1Z}^{M}\\ \vdots & {\hat{a}}_{ri}^{M} & \vdots & \vdots & -{\hat{b}}_{rr}^{M} & \vdots & \vdots & {\hat{c}}_{rz}^{M} & \vdots \\ {\hat{a}}_{R1}^{M} & \cdots & {\hat{a}}_{RI}^{M} & -{\hat{b}}_{R1}^{M} & \cdots & -{\hat{b}}_{RR}^{M} & {\hat{c}}_{R1}^{M} & \cdots & {\hat{c}}_{RZ}^{M}\\ {\hat{a}}_{11}^{L} & \cdots & {\hat{a}}_{1I}^{L} & -{\hat{b}}_{11}^{L} & \cdots & -{\hat{b}}_{1R}^{L} & {\hat{c}}_{11}^{L} & \cdots & {\hat{c}}_{1Z}^{L}\\ \vdots & {\hat{a}}_{zi}^{L} & \vdots & \vdots & -{\hat{b}}_{zr}^{L} & \vdots & \vdots & {\hat{c}}_{zz}^{L} & \vdots \\ {\hat{a}}_{Z1}^{L} & \cdots & {\hat{a}}_{ZI}^{L} & -{\hat{b}}_{Z1}^{L} & \cdots & -{\hat{b}}_{ZR}^{L} & {\hat{c}}_{Z1}^{L} & \cdots & {\hat{c}}_{ZZ}^{L} \end{array}\right]\in {\mathbb{R}}^{\left(I+K+R+Z)\times (I+R+Z\right)} \end{array}$$

where ${\hat{\alpha }}_{ij}$ denotes the interactive abilities of the *i*th protein with the *j*th protein in the PPIN which could be obtained from ${\hat{\theta }}_{i}^{P}$ by solving parameter estimation problem in Equation (6) and pruning the false positives by AIC in Equation (11); ${\hat{a}}_{ki}^{G}$, ${\hat{b}}_{kr}^{G}$, and ${\hat{c}}_{kz}^{G}$ represent transcriptional regulative abilities from the *i*th TFs, the *r*th miRNAs and the *z*th lncRNAs onto the *k*th protein-coding genes, respectively, which could be obtained from ${\hat{\theta }}_{k}^{G}$ by solving parameter estimation problem in Equation (10) and pruning the false positives by AIC in (12); ${\hat{a}}_{ri}^{M}$, ${\hat{b}}_{rr}^{M}$, and ${\hat{c}}_{rz}^{M}$ indicate the transcriptional regulative abilities from the *i*th TFs, the *r*th miRNAs and the *z*th lncRNAs onto the *r*th miRNA’s gene, respectively, which could be acquired from ${\hat{\theta }}_{r}^{M}$ by solving parameter estimation problem in Equation (S6) and pruning the false positives by AIC in Equation (S11); ${\hat{a}}_{zi}^{L}$, ${\hat{b}}_{zr}^{L}$, and ${\hat{c}}_{zz}^{L}$ indicate the transcriptional regulative abilities from the *i*th TFs, the *r*th miRNAs and the *z*th lncRNAs onto the *z*th lncRNA’s gene, respectively, which could be acquired from ${\hat{\theta }}_{z}^{L}$ by solving parameter estimation problem in Equation (S10) and pruning the false positives by AIC in Equation (S12). It is noted that if interactions or regulations do not exist in the candidate GWGEN via big data mining or already have been pruned by AIC, the corresponding components in matrix *H* are padded with zero.

As the H have been constructed, we thereby extract the core GWGEN from the real GWGEN by the PNP method shown as below. At first, the combined network matrix H can be a factorization of the following singular value decomposition (SVD) form as below:

(14) $$H=U\times D\times V^{T}$$

where $U\in {\mathbb{R}}^{\left(I+K+R+Z)\times (I+R+Z\right)}$, $V\in {\mathbb{R}}^{\left(I+R+Z)\times (I+R+Z\right)}$, and $D=\mathrm{diag}(d_{1},\cdots ,d_{I+R+Z})$. D is composed of I+R+Z singular values of H and $d_{1}\geq d_{2}\geq \cdots \geq d_{I+R+Z}$. The eigen expression fraction $E_{h}$ is defined in the following energy normalization:

(15) $$E_{h}=\frac{d_{h}^{2}}{\sum_{h=1}^{I+R+Z}d_{h}^{2}}$$

Then, we find out the minimum γ such that $\sum_{h=1}^{\gamma }E_{h}\geq 0.85$. That is, top γ singular vectors of matrix H contain 85% core network structure of the real GWGEN from the energy point of view. Additionally, we define the projections of H to the top γ singular vectors of V as

(16) $$N_{R}(w,s)=h_{w,:}\times v_{:,s}^{T}\;\mathrm{for}\;\;w=1,2,\ldots ,I^{*}+R^{*}+Z^{*}\;\mathrm{and}\;s=1,2,\ldots ,\gamma$$

where $h_{w:,}$ and $v_{:,s}^{T}$ denote the *w*th row of H and the *s*th column of V, respectively. Subsequently, for the top γ right-singular vectors, we define the 2-norm projection value of proteins, genes, lncRNAs, and miRNAs (i.e., the nodes) in the real GWGEN as below:

(17) $$D_{R}(w)={\left[\sum_{s=1}^{\gamma }{\left[N_{R}(w,s)\right]}^{2}\right]}^{1/2}\;\mathrm{for}\;\;w=1,2,\ldots ,I^{*}+R^{*}+Z^{*}\;\mathrm{and}\;s=1,2,\ldots ,\gamma$$

If the projection value, $D_{R}(w)$, approaches to zero for the *w*th node, it means that the wth node is almost independent to the principal network structure. That is, the larger the projection value is, the greater the contribution of the corresponding node to the core network is. By doing so, we can extract the core GWGEN by collecting nodes with large projection values from the real GWGENs and denote them in the KEGG pathway style to investigate the progression mechanisms of human skin aging.

### 4.7. Data Preprocessing for Training Deep Neural Network of Drug–Target Interaction in Advance

The drug–target interaction dataset comes from BindingDB [35]. The descriptors of drugs and targets are transformed by PyBioMed [112]. We install this package and import PyMolecule module and PyProtein module to transform drugs and targets into their descriptors under python 2.7 environment. The PyMolecule module in PyBioMed is responsible to compute the commonly used structural and physicochemical descriptors to be drug features. The drug features include constitutional and geometrical descriptors. Furthermore, the PyProtein module in PyBioMed is responsible for calculating the widely used descriptors, including structural and physicochemical properties of proteins and peptides from amino acid sequences, to be target features. Subseqently, concatenating the drug descriptor and the target descriptor, we describe properties of a drug and its target by a feature vector shown in (18). Moreover, the total number of drug features and target features are 363 and 996, respectively.

(18) $$v_{drug-t\arg et}=[D,T]=[d_{1},d_{2},\ldots ,d_{I},t_{1},t_{2},\ldots ,t_{J}]$$

where $v_{drug-t\arg et}$ indicates a feature vector of a drug-target pair; D denotes the feature vector of the drug; $d_{i}$ indicates the *i*th drug feature; T represents the feature vector of the target; $t_{j}$ is the *j*th target feature; *I* is the total number of drug features; *J* is the total number of target features. We conduct the same transformation for all the drug-target pairs to obtain their drug-target feature vectors.

## Figures and Tables

**Table 1:** The table of the total number of nodes and edges in candidate GWGENs and identified real GWGENs for each stage of skin aging.

	Candidate GWGEN	Young-adult GWGEN	Middle-aged GWGEN	Elderly GWGEN

TFs	1851	464	491	383
TF—protein	429,829	11,715	10,888	10,234
TF—receptor	66,042	1824	1738	1639
TF—TF	27,892	513	468	223
TF—lncRNAs	1600	494	457	473
TF—miRNA	348	84	76	91
lncRNAs	666	593	522	582
lncRNAs—protein	19,520	1944	1902	2286
lncRNAs—TF	1443	88	90	84
lncRNAs—receptor	3018	293	288	366
miRNAs	126	111	99	107
miRNA—protein	45,613	4175	3997	4884
miRNA—receptor	6891	593	707	745
miRNA—TF	3028	782	680	849
Receptors	2388	2372	2366	2387
Proteins	15,347	13,195	13,219	13,213
PPIs	3,185,763	172,558	146,594	97,766
Total nodes	20,378	16,735	16,697	16,672
Total edges	3,790,987	195,063	167,885	119,640

**Table 2:** Drug targets and multiple-molecule drugs for preventing skin aging from young adulthood to middle-age.

Target	AIFM1	CAT	IGF1R	LMNA
Drug				
niridazole	●	●		●
liothyronine	●			
decitabine		●		
pinacidil			●	
allantoin			●	

●: Proposed small molecules target to the identified biomarkers (drug targets).

**Table 3:** Drug targets and multiple-molecule drugs for preventing skin aging from middle age to old age.

Target	MMP9	IL6	BCL2	CASP3
Drug				
allantoin	●	●		
diclofenac	●			
mepyramine	●		●	
resveratrol			●	●
azathioprine			●	●

●: Proposed small molecules target to the identified biomarkers (drug targets).

## Data Availability

The human skin data is from GSE18876 (https://www.ncbi.nlm.nih.gov/geo/query/acc.cgi?acc=GSE18876) (accessed on 19 May 2021). Drug sensitivity data is from depmap portal (https://depmap.org/portal/download/) (accessed on 19 May 2021).

## Supplementary Materials

The following are available online, Figure S1: The real genome-wide genetic and epigenetic network (GWGEN) of young-stage skin, Figure S2: The real genome-wide genetic and epigenetic network (GWGEN) of middle-stage skin, Figure S3: The real genome-wide genetic and epigenetic network (GWGEN) of elder-stage skin, Figure S4: Deep neural network of drug-target interaction framework, Table S1: The pathway enrichment analysis of proteins through applying the DAVID in the core GWGEN of young-stage skin, Table S2: The pathway enrichment analysis of proteins through applying the DAVID in the core GWGEN of middle-stage skin, Table S3: The pathway enrichment analysis of proteins through applying the DAVID in the core GWGEN of elder-stage skin, Table S4: Drug targets with their corresponding small-molecule compounds, Table S5: Drug targets with their corresponding small-molecule compounds.
