# Supplementary material for: Multiple-Molecule Drug Design Based on Systems Biology Approaches and Deep Neural Network to Mitigate Human Skin Aging
Source: Molecules. 2021 May 26;26(11):3178. doi: 10.3390/molecules26113178 (PMC8197996; doi:10.3390/molecules26113178)
Supplement: Supplementary file 1 [file molecules-26-03178-s001.zip › molecules-1223612-supplementary.pdf]

Supplementary Materials.

1.1 Dynamic systems modeling for the candidate GWGEN

For the candidate miRNA regulation network in the candidate GWGEN, the *r*th miRNA can be described by a stochastic dynamic equation as below:

$$
\begin{aligned}
m_r(t+1) = {} & m_r(t) + \sum_{i=1}^{I_r} a_{ri}^M p_i(t) - \sum_{n=1}^{R_r} b_{rn}^M m_r(t) m_n(t) + \sum_{\ell=1}^{L_r} c_{r\ell}^M o_\ell(t) \\
& -\mu_r^M m_r(t) + \delta_r^M + \omega_r^M(t), \\
& for\ r = 1, 2 \ldots, R, \quad -b_{rn}^M \le 0\ and\ -\mu_r^M \le 0
\end{aligned}
\qquad \text{(s1)}
$$

where R is the total number of miRNA in candidate GWGEN; $m_r(t)$, $p_i(t)$, and $o_\ell(t)$ represent the expression level of the rth miRNA, the ith TF, and the ℓ th lncRNA, respectively; a_{ri}^M, $-b_{rn}^M$, and $c_{r\ell}^M$ signify the regulatory abilities of the ith TF regulation, the nth miRNA repression, and the ℓ th lncRNA regulation, respectively; $-\mu_r^M$ and δ_r^M denote the degradation rate and basal level of the rth miRNA, respectively; $\omega_r^M(t)$ represents the stochastic noise of the rth miRNA at time t. Note that the biological regulatory mechanisms in equation (s1) involve TF transcription regulations by $\sum_{i=1}^{I_r} a_{ri}^M p_i(t)$, miRNA repressions by $-\sum_{n=1}^{R_r} b_{rn}^M m_r(t) m_n(t)$, lncRNA regulations by $\sum_{\ell=1}^{L_r} c_{r\ell}^M o_\ell(t)$, the miRNA degradation by $-\mu_r^M m_r(t)$, the basal level by δ_r^M, and the stochastic noise by $\omega_r^M(t)$.

For the candidate lncRNA regulation network in the candidate GWGEN, the *z*th lncRNA can be described in a stochastic dynamic equation as bellow:

$$
\begin{aligned}
o_z(t+1) = {} & o_z(t) + \sum_{i=1}^{I_z} a_{zi}^L p_i(t) - \sum_{r=1}^{R_z} b_{zr}^L o_z(t) m_r(t) + \sum_{x=1}^{L_z} c_{zx}^L o_x(t) \\
& -\mu_z^L o_z(t) + \delta_z^L + \omega_z^L(t), \\
& for\ z = 1, 2 \ldots, Z, \quad -b_{zr}^L \le 0\ and\ -\mu_z^L \le 0
\end{aligned}
\qquad \text{(s2)}
$$

where Z is the total number of lncRNA in candidate GWGEN; $o_z(t)$, $p_i(t)$, $m_r(t)$, and $\omega_z^L(t)$

represent the expression level of the zth lncRNA, the ith TF, the rth miRNA, and stochastic noise at time t, respectively; a_{zi}^{L}, $-b_{zr}^{L}$, and c_{zx}^{L} signify the regulatory abilities of the ith TF regulation, the rth miRNA repression, and the xth lncRNA regulation, respectively; $-\mu_{z}^{L}$ and δ_{z}^{L} denote the degradation rate and basal level of the zth lncRNA, respectively. Note that the biological regulatory mechanisms in equation (s2) involve TF transcription regulations by $\sum_{i=1}^{I_z} a_{zi}^{L} p_i(t)$, miRNA repressions by $-\sum_{n=1}^{R_z} b_{zn}^{L} o_z(t) m_n(t)$, lncRNA regulations by $\sum_{x=1}^{L_z} c_{zx}^{L} o_x(t)$, the lncRNA degradation by $-\mu_z^L m_z(t)$, the basal level by δ_z^L, and the stochastic noise by $\omega_z^L(t)$.

1.2 Systems identification approach in the candidate GWGEN via microarray data

The dynamic equation for miRNA in equation (s1) can be rewrite in the following liner regression form:

$$
\begin{aligned}
m_r(t+1) = [\quad & p_1(t)\cdots p_{I_r}(t) \quad m_r(t)m_1(t)\cdots m_r(t)m_{R_r}(t) \\
& o_1(t)\cdots o_{L_r}(t) \quad m_r(t) \quad 1]
\begin{bmatrix} a_{r_1}^{M} \\ \vdots \\ a_{rI_r}^{M} \\ -b_{r_1}^{M} \\ \vdots \\ -b_{rR_r}^{M} \\ c_{r_1}^{M} \\ \vdots \\ c_{rL_r}^{M} \\ 1-\mu_r^{M} \\ \delta_r^{M} \end{bmatrix} + \omega_r^M(t), \\
& = \psi_r^M(t)\theta_r^M + w_r^M(t), \quad for\ r = 1,2,\ldots,R
\end{aligned} \tag{s3}
$$

where $\psi_r^M(t)$ represents the regression vector that can be obtained from the microarray data and θ_r^M signifies the unknown parameter vector to be estimated for the rth miRNA in the miRNA regulation network. By observing the equation (s3), the rth miRNA for Y_r time points can be rewritten as the following form:

$$\begin{bmatrix} m_r(t_2) \\ m_r(t_3) \\ \vdots \\ m_r(t_{Y_r}+1) \end{bmatrix} = \begin{bmatrix} \psi_r^M(t_1) \\ \psi_r^M(t_2) \\ \vdots \\ \psi_r^M(t_{Y_r}) \end{bmatrix} \theta_r^M + \begin{bmatrix} \omega_r^M(t_1) \\ \omega_r^M(t_2) \\ \vdots \\ \omega_r^M(t_{Y_r}) \end{bmatrix}, \quad \text{for } r = 1, 2, \ldots, R \tag{s4}$$

Next, we simplify the equation (s4) in the form shown below:

$$M_r = \Psi_r^M \theta_r^M + \Omega_r^M, \quad \text{for} = 1, 2, \ldots, R \tag{s5}$$

where

$$M_r = \begin{bmatrix} m_r(t_2) \\ m_r(t_3) \\ \vdots \\ m_r(t_{Y_r}+1) \end{bmatrix}, \quad \Psi_r^M = \begin{bmatrix} \psi_r^M(t_1) \\ \psi_r^M(t_2) \\ \vdots \\ \psi_r^M(t_{Y_r}) \end{bmatrix}, \quad \Omega_r^M = \begin{bmatrix} \omega_r^M(t_1) \\ \omega_r^M(t_2) \\ \vdots \\ \omega_r^M(t_{Y_r}) \end{bmatrix}.$$

Therefore, the regulatory parameters in the vector θ_r^M can be estimated by solving the following constrained least-squares estimation problem:

$$\hat{\theta}_r^M = \min_{\theta_r^M} \frac{1}{2} \left\| \Psi_r^M \theta_r^M - M_r \right\|_2^2, \quad \text{subject to} \quad A^M \theta_r^M \le b^M \tag{s6}$$

where

$$A^R = \begin{bmatrix} 0 & 0 & \cdots & 0 & 1 & 0 & \cdots & 0 & 0 & 0 & \cdots & 0 & 0 & 0 \\ 0 & 0 & \cdots & 0 & 0 & 1 & \cdots & 0 & 0 & 0 & \cdots & 0 & 0 & 0 \\ \vdots & \vdots & \ddots & \vdots & \vdots & \vdots & \ddots & \vdots & \vdots & \vdots & \ddots & \vdots & \vdots & \vdots \\ 0 & 0 & \cdots & 0 & 0 & 0 & \cdots & 1 & 0 & 0 & \cdots & 0 & 0 & 0 \\ 0 & 0 & \cdots & 0 & 0 & 0 & \cdots & 0 & 0 & 0 & \cdots & 0 & 1 & 0 \end{bmatrix} \in \mathbb{R}^{(R_r+1)\times(I_r+R_r+L_r+2)}$$

$$, \quad b^M = \begin{bmatrix} 0 \\ \vdots \\ 1 \end{bmatrix}.$$

By applying the function $lsqlin$ in MATLAB optimization toolbox to solve the parameter estimation problem in equation (s6), we can estimate the regulatory parameters in equation (s1). Furthermore, we

ensure that the miRNA repression ability $-b_{rn}^{M}$ to be a non-positive value and the miRNA degradation rate $-\mu_{r}^{M}$ to be a non-positive value for $r=1,2,\ldots R$ and $n=1,2,\ldots R_r$.

Similarly, the *z*th lncRNA dynamic regulation in equation (s2) can be written in the following linear regression form:

$$\begin{aligned} o_z(t+1) &= [\ p_1(t)\cdots p_{I_z}(t) \quad o_z(t)m_1(t)\cdots o_z(t)m_{R_z}(t) \\ &\quad o_1(t)\cdots o_{L_z}(t) \quad o_z(t) \quad 1\,] \begin{bmatrix} a_{z_1}^{L} \\ \vdots \\ a_{zI_z}^{L} \\ -b_{z_1}^{L} \\ \vdots \\ -b_{zR_z}^{L} \\ c_{z_1}^{L} \\ \vdots \\ c_{zL_z}^{L} \\ 1-\mu_z^{L} \\ \delta_z^{L} \end{bmatrix} + \omega_z^{L}(t), \\ &= \psi_z^{L}(t)\theta_z^{L} + w_z^{L}(t), \quad for\ z=1,2,\ldots,Z \end{aligned} \tag{s7}$$

where $\psi_z^{L}(t)$ represents the regression vector that can be obtained from the microarray data and θ_z^{L} signifies the unknown parameter vector to be estimated for the zth lncRNA in equation (s2). By observing the equation (s7), the zth lncRNA for Y_z time points can be rewritten as

$$\begin{bmatrix} o_z(t_2) \\ o_z(t_3) \\ \vdots \\ o_z(t_{Y_z}+1) \end{bmatrix} = \begin{bmatrix} \psi_z^{L}(t_1) \\ \psi_z^{L}(t_2) \\ \vdots \\ \psi_z^{L}(t_{Y_z}) \end{bmatrix} \theta_r^{M} + \begin{bmatrix} \omega_z^{L}(t_1) \\ \omega_z^{L}(t_2) \\ \vdots \\ \omega_z^{L}(t_{Y_z}) \end{bmatrix}, \quad for\ z=1,2,\ldots,Z \tag{s8}$$

Afterwards, we simplify the equation (s8) as below:

$$O_z = \Psi_z^{L}\theta_z^{L} + \Omega_z^{L}, \quad \text{for z} = 1,2,\ldots,Z \tag{s9}$$

where $O_z = \begin{bmatrix} o_z(t_2) \\ o_z(t_3) \\ \vdots \\ o_z(t_{Y_z}+1) \end{bmatrix}$, $\Psi_z^L = \begin{bmatrix} \psi_z^L(t_1) \\ \psi_z^L(t_2) \\ \vdots \\ \psi_z^L(t_{Y_z}) \end{bmatrix}$, $\Omega_z^L = \begin{bmatrix} \omega_z^L(t_1) \\ \omega_z^L(t_2) \\ \vdots \\ \omega_z^L(t_{Y_z}) \end{bmatrix}$.

Hence, the regulatory parameters in the vector θ_z^L can be estimated by solving the following constrained least-squares estimation problem:

$$\hat{\theta}_z^L = \min_{\theta_z^L} \frac{1}{2} \left\| \Psi_z^L \theta_z^L - O_z \right\|_2^2, \quad \text{subject to} \quad A^L \theta_z^L \le b^L \tag{s10}$$

where

$$A^L = \begin{bmatrix} 0 & 0 & \cdots & 0 & 1 & 0 & \cdots & 0 & 0 & 0 & \cdots & 0 & 0 & 0 \\ 0 & 0 & \cdots & 0 & 0 & 1 & \cdots & 0 & 0 & 0 & \cdots & 0 & 0 & 0 \\ \vdots & \vdots & \ddots & \vdots & \vdots & \vdots & \ddots & \vdots & \vdots & \vdots & \ddots & \vdots & \vdots & \vdots \\ 0 & 0 & \cdots & 0 & 0 & 0 & \cdots & 1 & 0 & 0 & \cdots & 0 & 0 & 0 \\ 0 & 0 & \cdots & 0 & 0 & 0 & \cdots & 0 & 0 & 0 & \cdots & 0 & 1 & 0 \end{bmatrix} \in \mathbb{R}^{(R_z+1)\times(I_z+R_z+L_z+2)}$$

, $b^L = \begin{bmatrix} 0 \\ \vdots \\ 1 \end{bmatrix}$.

Applying the function $lsqlin$ in MATLAB optimization toolbox to solve the parameter estimation problem in equation (s10), we can estimate the regulatory parameters in equation (s2). Furthermore, we ensure that the miRNA repression ability $-b_{zr}^L$ to be a non-positive value and the lncRNA degradation rate $-\mu_z^L$ to be a non-positive value for $r = 1, 2, \ldots R_z$.

1.3 Pruning false-positives in candidate GWGENs to obtain real GWGENs by system order detection scheme

For the miRNA model in equation (s5), AIC value of the *r*th miRNA can be defined in the following equation:

$$AIC_r^M(I_r, R_r, L_r) = \log\left\{\frac{1}{T_r}\left[M_r - \Psi_r^M \hat{\theta}_r^M\right]^T \left[M_r - \Psi_r^M \hat{\theta}_r^M\right]\right\} + \frac{2(I_r + R_r + L_r)}{T_r} \quad \text{(s11)}$$

where $\hat{\theta}_r^M$ denotes the estimated interactive parameters of the *r*th miRNA from the solutions of the parameter estimation problem in equation (s6), and the covariance of estimated residual error is $(\varsigma_r^M)^2 = \frac{1}{T_r}\left[M_r - \Psi_r^M \hat{\theta}_r^M\right]^T \left[M_r - \Psi_r^M \hat{\theta}_r^M\right]$. In order to find out the real system order I_r^*, R_r^*, and O_r^* of the real regulations of the *r*th miRNA in GRN, we have to minimize $AIC_r^M(I^*_r, R^*_r, L^*_r)$ in equation (s11). By the system order detection scheme, miRNAs with insignificant repression abilities, which are out of I_r^*, R_r^*, and O_r^* can be regarded as false-positives and pruned away from the candidate miRNA GRN of the *r*th miRNA.

For the lncRNA model in equation (s9), AIC value of the *z*th lncRNA can be defined as below:

$$AIC_z^L(I_z, R_z, L_z) = \log\left\{\frac{1}{T_z}\left[O_z - \Psi_z^L \hat{\theta}_z^L\right]^T \left[O_z - \Psi_z^L \hat{\theta}_z^L\right]\right\} + \frac{2(I_z + R_z + L_z)}{T_z} \quad \text{(s12)}$$

where $\hat{\theta}_z^L$ denotes the estimated interactive parameters of the *z*th lncRNA from the solutions of the parameter estimation problem in equation (s10), and the covariance of estimated residual error is $(\varsigma_z^L)^2 = \frac{1}{T_z}\left[O_z - \Psi_z^L \hat{\theta}_z^L\right]^T \left[O_z - \Psi_z^L \hat{\theta}_z^L\right]$. In order to find out the real system order I_z^*, R_z^*, and O_z^* of the zth lncRNA, we minimize $AIC_z^L(I_z, R_z, L_z)$ in equation (s12). By the system order detection scheme, lncRNAs with insignificant interaction abilities, which are out of I_z^*, R_z^*, and O_z^*, can be treated as false-positives and pruned away from the candidate lncRNA GRN of the *z*th lncRNA.

Tables

Table S1. The pathway enrichment analysis of proteins through applying the DAVID in the core GWGEN of young-stage skin.

Term	Numbers	p-value
Pathways in cancer	91	4.33E-04
HTLV-I infection	62	1.06E-03
MAPK signaling pathway	61	1.60E-03
Viral carcinogenesis	60	5.42E-06
Endocytosis	60	7.70E-04

Table S2. The pathway enrichment analysis of proteins through applying the DAVID in the core GWGEN of middle-stage skin.

Term	Numbers	p-value
Pathways in cancer	99	6.64E-06
PI3K-Akt signaling pathway	72	2.34E-02
Viral carcinogenesis	67	1.40E-08
HTLV-1 infection	65	1.99E-04
Alcoholism	52	2.26E-05

Table S3. The pathway enrichment analysis of proteins through applying the DAVID in the core GWGEN of elder-stage skin

Term	Numbers	p-value
Pathways in cancer	107	1.40E-07
PI3K-Akt signaling pathway	75	1.36-02
Viral carcinogenesis	67	3.53E-08
Alcoholism	61	1.81E-08
MAPK signaling pathway	59	7.01E-03

Table S4. Drug targets with their corresponding small-molecule compounds

AIFM1 (-)			CAT (-)		
Drug	Perturbation Signature	Sensitivity	Drug	Perturbation Signature	Sensitivity
ranitidine	0.003	0.129	niridazole	0.064	0.052
niridazole	0.02	0.052	decitabine	0.072	-1.186
liothyronine	0.042	-0.006			
**IGF1R (+)**			**LMNA (-)**		
Drug	Perturbation Signature	Sensitivity	Drug	Perturbation Signature	Sensitivity
pinacidil	-0.061	-0.103	niridazole	0.052	0.052
allantoin	-0.075	-0.053			

+ abnormal upregulation

-abnormal downregulation

Table S5. Drug targets with their corresponding small-molecule compounds

MMP9 (+)			IL6 (-)		
Drug	Perturbation Signature	Sensitivity	Drug	Perturbation Signature	Sensitivity
allantoin	-0.137	-0.053	allantoin	0.016	-0.053
diclofenac	-0.086	-0.232			
mepyramine	-0.021	-0.037			

BCL2 (+)			CASP3 (+)		
Drug	Perturbation Signature	Sensitivity	Drug	Perturbation Signature	Sensitivity
resveratrol	-0.685	-0.305	resveratrol	-0.771	-0.305
mepyramine	-0.043	-0.037	azathioprine	-0.393	-1.053
azathioprine	-0.347	-1.053			

+ *abnormal upregulation*

-*abnormal downregulation*

Figures

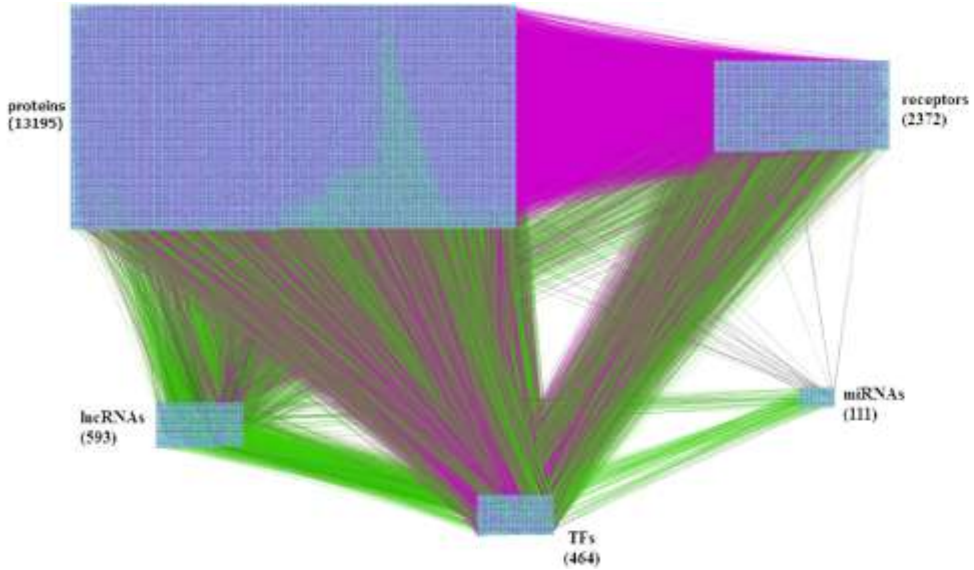

Figure S1. The real genome-wide genetic and epigenetic network (GWGEN) of young-stage skin. The purple lines denote protein-protein interactions (PPIs); The green lines indicate transcriptional regulations by TFs and lncRNAs; The black lines represent post-transcriptional regulations by miNRAs; The numbers of Receptors, Proteins, lncRNAs, TFs and miRNAs are 2372, 14941, 593, 464 and 111, respectively.

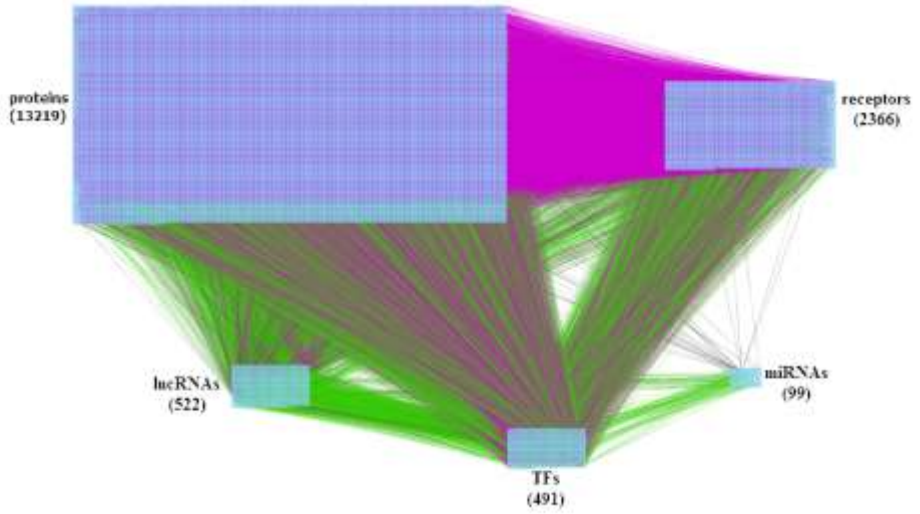

Figure S2. The real genome-wide genetic and epigenetic network (GWGEN) of middle-stage skin. The purple lines denote protein-protein interactions (PPIs); The green lines indicate transcriptional regulations by TFs and lncRNAs; The black lines represent post-transcriptional regulations by miNRAs; The numbers of Receptors, Proteins, lncRNAs, TFs and miRNAs are 2366, 14910, 522, 491 and 99, respectively.

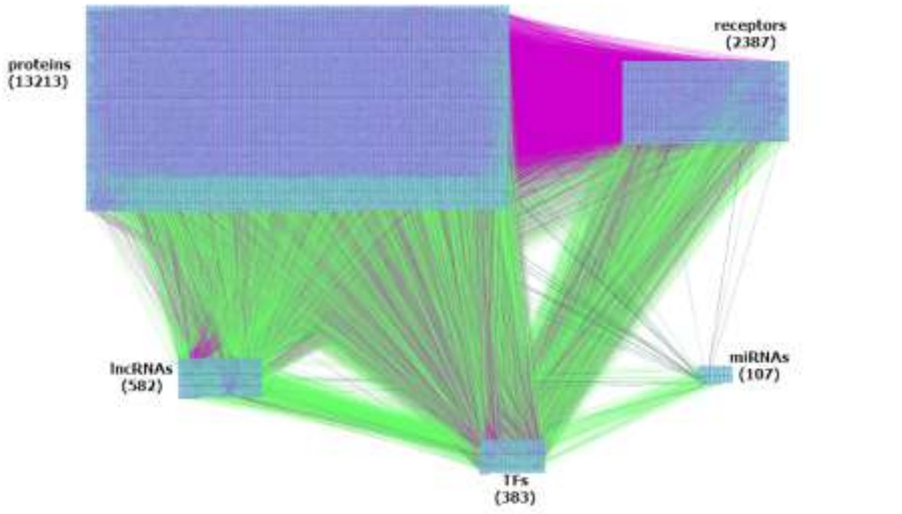

Figure S3. The real genome-wide genetic and epigenetic network (GWGEN) of elder-stage skin. The purple lines denote protein-protein interactions (PPIs); The green lines indicate transcriptional regulations by TFs and lncRNAs; The black lines represent post-transcriptional regulations by miNRAs; The numbers of Receptors, Proteins, lncRNAs, TFs and miRNAs are 2387, 13213, 582, 383 and 107, respectively.

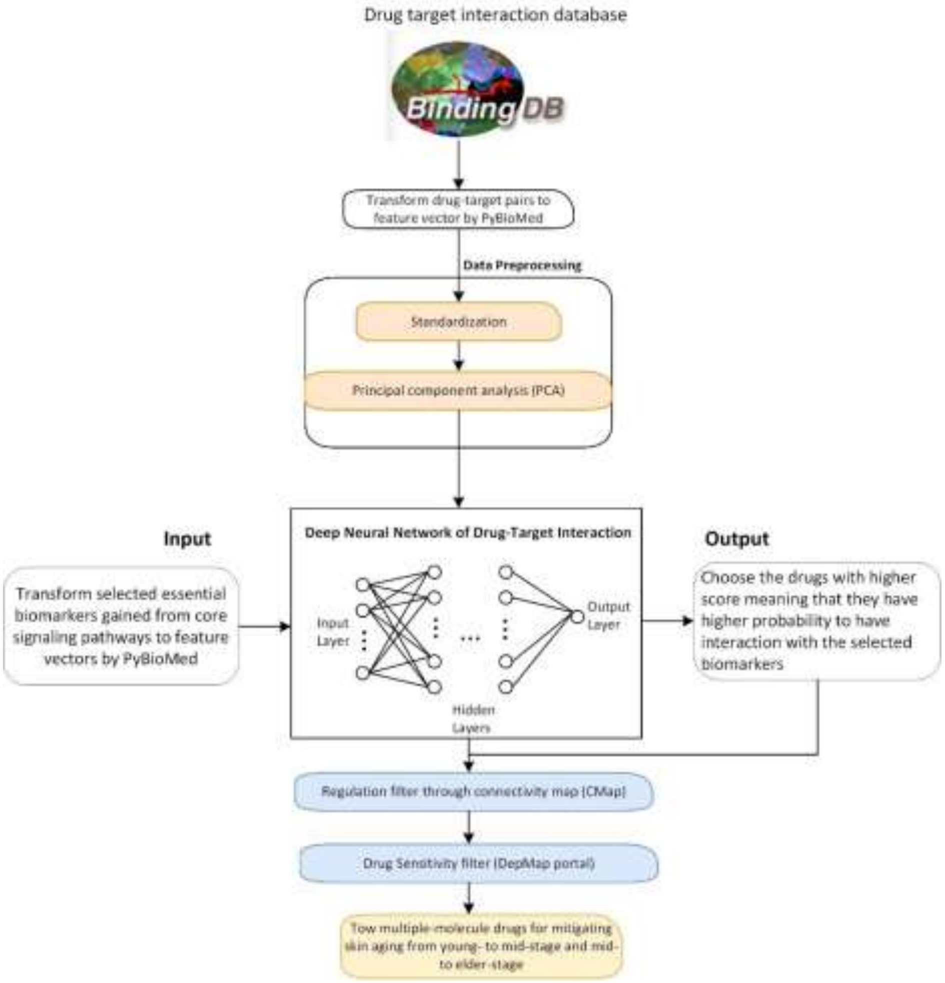

Figure S4. Deep neural network of drug-target interaction framework.
